# Dysfunction of α2δ4 leads to photoreceptor degeneration through disrupted synaptic mitochondria and calcium crosstalk

**DOI:** 10.1038/s41419-026-08587-3

**Published:** 2026-03-23

**Authors:** Choice I. Amieghemen, Trong Thuan Ung, Gillian N. Huskin, James A. Mobley, Melissa F. Chimento, Mai Nguyen, James Fortenberry, Timothy W. Kraft, Steven J. Pittler, Yuchen Wang

**Affiliations:** 1 Department of Optometry and Vision Science, The University of Alabama at Birmingham, Birmingham, AL USA; 2 Department of Anesthesiology and Perioperative Medicine, Division of Molecular and Translational Biomedicine, The University of Alabama at Birmingham, Birmingham, AL USA; 3 Institutional Research Core Program, High Resolution Imaging Facility, The University of Alabama at Birmingham, Birmingham, AL USA

**Keywords:** Ion channels in the nervous system, Mechanisms of disease, Retina, Molecular neuroscience, Calcium channels

## Abstract

Synaptic deficit has emerged as a key early hallmark for neurodegeneration in the visual pathway. The molecular pathway connecting local synaptic deficit with global cell dysfunction and death remains unclear. We have previously shown that α2δ4, an auxiliary subunit of the voltage-gated calcium channel, is targeted to photoreceptor synapses and required for their formation and function. Notably, α2δ4 mutations have been identified in patients with retinal dystrophy. However, how loss of synaptic α2δ4 leads to overall photoreceptor degeneration remains unknown. Here, we showed that α2δ4 loss in mice leads to a late onset photoreceptor degeneration around 7 months. Consistent with clinical observation, the progression of degeneration is minimal until 17 months, as supported by ERG, OCT imaging and histology. We found that Cav1.4 KO mice, where the calcium channel is missing, display an earlier degeneration onset than α2δ4 KO mice, where calcium channel is partially preserved. Proteomic studies revealed that tricarboxylic acid (TCA) cycle is significantly downregulated in the young α2δ4 KO retinas prior to degeneration. Transmission electron microscopy study demonstrated significant reduction in mitochondrial size and number in photoreceptor synaptic terminals, but not in the inner segment (IS), of the young α2δ4 KO retinas. Consistently, immunohistochemistry (IHC) studies showed significant reduction of mitochondrial proteins in the outer plexiform layer (OPL). IHC studies on ER and mitochondrial proteins revealed that ryanodine receptor (RyR2) and mitochondrial calcium uniporter (MCU) are downregulated in the OPL, but not in the IS. Together, our results propose a model where α2δ4 dysfunction impairs Cav1.4 channel activity, leading to disrupted calcium crosstalk among the plasma membrane, ER, and mitochondria, as well as mitochondrial damage and metabolic deficits. Importantly, our study underscores the critical role of synaptic calcium homeostasis and mitochondrial integrity in connecting the early stages of synaptic dysfunction with the later stages of cell degeneration.

## Introduction

Retinal degeneration is the leading cause for irreversible blindness [1]. It covers an array of retinal diseases, including inherited retinal diseases (IRDs) such as retinitis pigmentosa (RP), age-related macular degeneration (AMD), diabetic retinopathy, and glaucoma. Despite their prevalence, there is no cure for most forms due to an incomplete understanding of the relevant molecular changes during degeneration, particularly those occurring at the early stage, when intervention would be most effective. Emerging evidence has indicated that synaptic remodeling in both the outer and inner retina is one early hallmark common for almost all retinal degenerations [2–12]. In the case of photoreceptor degeneration in both patients and animal models, synaptic remodeling is observed prior to significant cell loss in the form of bipolar cell dendrites extending over the outer plexiform layer (OPL) accompanied by photoreceptor axons retracting into the outer nuclear layer (ONL) [6, 13]. Understanding whether and how synaptic alteration contributes to retinal neuron death thus holds the promise to reveal the mechanism underlying the early stages of retinal degeneration.

α2δ proteins have been known as the extracellular auxiliary subunits of the voltage-gated calcium channels (VGCCs). They are required for the membrane trafficking of the pore-forming subunit α1 and critical for the voltage-dependent gating of VGCCs [14–16]. Among the four α2δ isoforms (α2δ1-4), α2δ1-3 are broadly expressed in the brain and other tissues, while α2δ4 is preferentially enriched in the retina, particularly in both rod and cone photoreceptors [17–21]. α2δ4 has been shown as the auxiliary subunit of the Cav1.4 complex which also consists of the pore-forming subunit α1f (CACNA1F) and the intracellular auxiliary subunit β2 (CACNB2) [22, 23]. The Cav1.4 complex is localized at the photoreceptor synaptic ribbons and controls graded glutamate release into the synaptic cleft by regulating the Ca^2+^ influx into photoreceptor terminals [19, 21, 24, 25].

Mutations in α2δ4 have been identified in patients displaying severely impaired ERG b-wave, which reflects the disruption of signal transmission from photoreceptors to bipolar cells (BCs), a hallmark of congenital stationary night blindness (CSNB) [26–30]. In addition, progressive decline of visual function was also observed in patients carrying α2δ4 mutations, indicating photoreceptor atrophy [26, 30]. In line with this, animal studies on α2δ4 have shown that loss of α2δ4 leads to disrupted synaptic function and organization for both rod and cone photoreceptors [19, 21, 31–33]. Interestingly, no overall retinal degeneration was observed in α2δ4 KO mice till 6 months [19, 21]. Despite the extensive studies of α2δ4 in the context of synapses, much less is known about the role of α2δ4 in photoreceptor degeneration. Answering this question will provide novel insights into photoreceptor degeneration, particularly the early stages of the process.

Here, we conducted a longitudinal investigation of the structural, functional and molecular changes in the retinas of α2δ4 KO mice. We found that loss of α2δ4 leads to a late onset photoreceptor degeneration around 7 months and that the degeneration progresses minimally within the time span of 17 months. We compared the functional and structural changes of α2δ4 KO retina with those of age-matched Cav1.4 KO retinas and found that the degeneration onset and extent in α2δ4 KO retinas are slightly slower and milder compared to Cav1.4 KO retina, consistent with the degrees of calcium channel dysfunction in these two models. Quantitative proteomics using young (2-month-old) retinas revealed tricarboxylic acid (TCA) cycle as the top disrupted signaling pathway. Consistently, light and electron microscopy studies demonstrated significant reduction in mitochondrial protein content and mitochondrial size and number in photoreceptor axonal terminals, but not in the inner segment (IS), of the young α2δ4 KO retinas. Quantitative IHC studies on the ER calcium channels and mitochondrial calcium transporter showed downregulation of RYR2 and MCU specifically in the OPL of young α2δ4 KO retinas. Together, our studies identified mitochondria damage and disrupted Ca^2+^ crosstalk within the photoreceptor terminals as two early cellular changes before the onset of photoreceptor degeneration in α2δ4 KO retinas. These findings suggest a model where local synaptic deficits trigger overall retinal neuron death by first disrupting local metabolism and calcium signaling.

## Results

### Loss of α2δ4 leads to late onset and mild photoreceptor degeneration

**Fig. 1:**
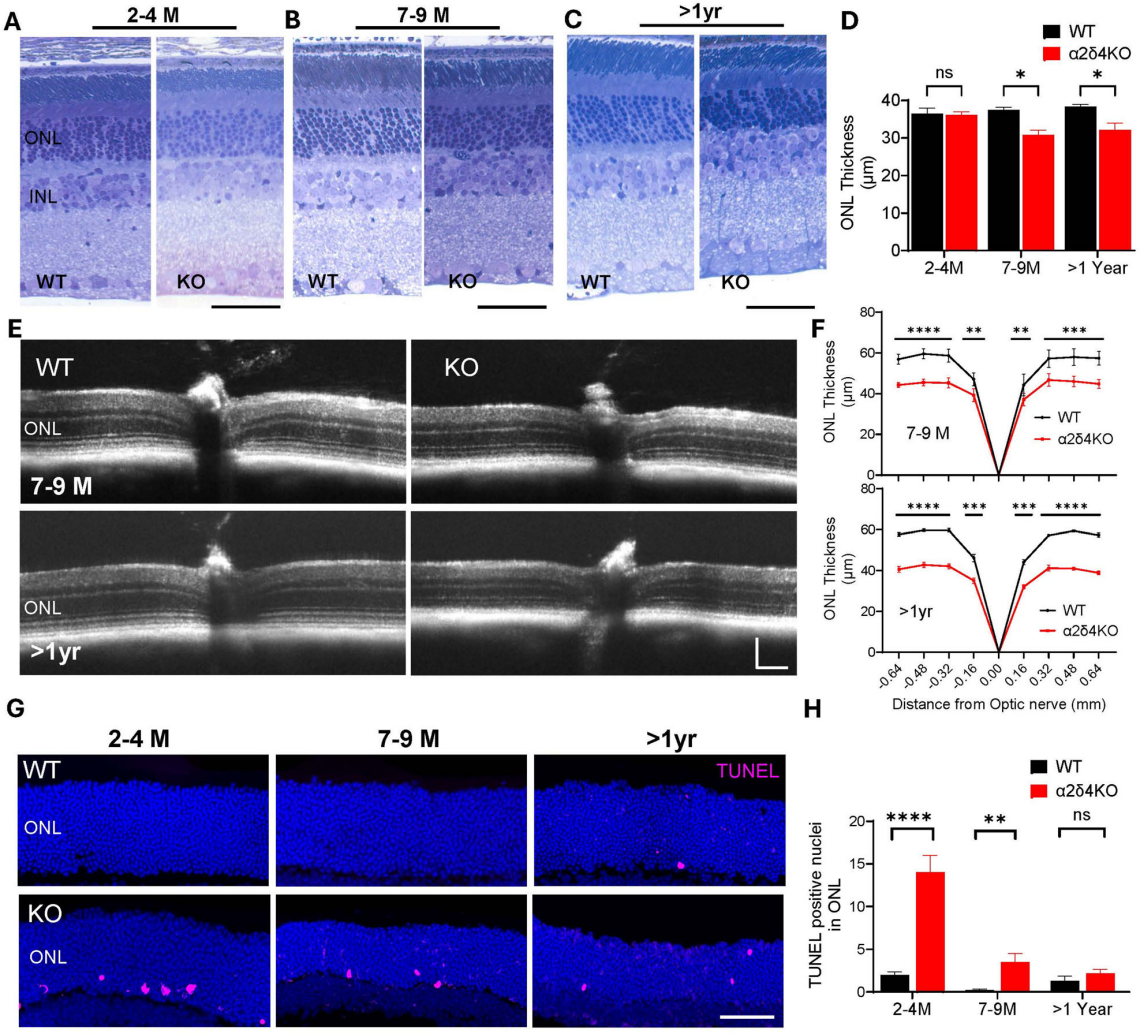
Histology, OCT imaging, and TUNEL labeling reveal age-dependent photoreceptor degeneration in α2δ4KO mice. **A**–**C** Representative images of toluidine blue-stained retinal sections from wild-type (WT) and α2δ4 knockout (KO) mice at (**A**). 2–4 months, **B** 7–9 months, and **C**. >1 year. The outer nuclear layer (ONL) and inner nuclear layer (INL) are labeled. Scale bar, 50 μm. **D** Quantification of central ONL thickness in WT and KO mice (*n* = 3 mice per group). **E** Representative optical coherence tomography (OCT) images of WT and KO retina at 7–9 months and > 1 year. Scale bar, 100 μm. **F** Quantification of ONL thickness across retinal eccentricities in 7–9 month (*top*) and >1 year (*bottom*) WT and KO retina (*n* = 8 mice per group). **G** Representative images of TUNEL-stained retinal cross-sections showing apoptotic photoreceptors (*pink*) in WT and KO mice at 2–4 months, 7–9 months, and >1 year. **H** Quantificatsion of number of TUNEL-positive cells in the ONL across the full retina section in WT and KO mice (*n* = 4 per group). Error bars are SEM, unpaired t-test. *p < 0.01, ***p < 0.001, ****p < *0.0001, ns; not significant*.

Since loss of α2δ4 in mice does not lead to overall retinal morphology change in young animals [19, 21, 32], we thus examined the impact of α2δ4 loss on retinal structure at later stages (7–9 and 15–17 months) using previously characterized α2δ4 KO mice [19]. We first assessed the retinal layer thickness using toluidine blue (TB) staining, followed by measurement of all major layers of the retina. We confirmed that the outer nuclear layer (ONL), where photoreceptor cell bodies are located is comparable between WT and KO retinas at 2–4 months (Fig. 1A, D). In contrast, the ONL of α2δ4 KO retinas became significantly thinner by ~20% at 7–9 months, and this difference in ONL thickness remained relatively unchanged till 15–17 months (>1 yr) (Fig. 1B–D). We also measured the thickness of the inner nucleus layer (INL) and inner plexiform layer (IPL) and found no significant change between genotypes at all three time points (Supplementary Fig. 1A, B). To confirm the histology results, we performed in vivo OCT imaging followed by comparing the ONL thickness at different eccentricities. Consistent with TB staining, we found that the thickness of ONL in the 7–9 month- and >1yr-old KO mice was significantly reduced while the thickness of INL remained unchanged (Fig. 1E, F; Supplementary Fig. 1C, D).

To confirm that the thinning of ONL is due to photoreceptor death, we performed TUNEL assay on retina sections from both WT and α2δ4 KO animals. Interestingly, we observed significantly more TUNEL positive cells in α2δ4 KO retina even at 2–4 months, when photoreceptor degeneration was not readily detected. The number of the apoptotic cells in KO retinas dropped in the 7–9 months group and became comparable between genotypes in >1 yr group (Fig. 1G, H).

Together, our data demonstrated that loss of α2δ4 leads to an early onset apoptosis but a late onset and mild photoreceptor degeneration.

### Loss of α2δ4 impairs overall photoreceptor function

**Fig. 2:**
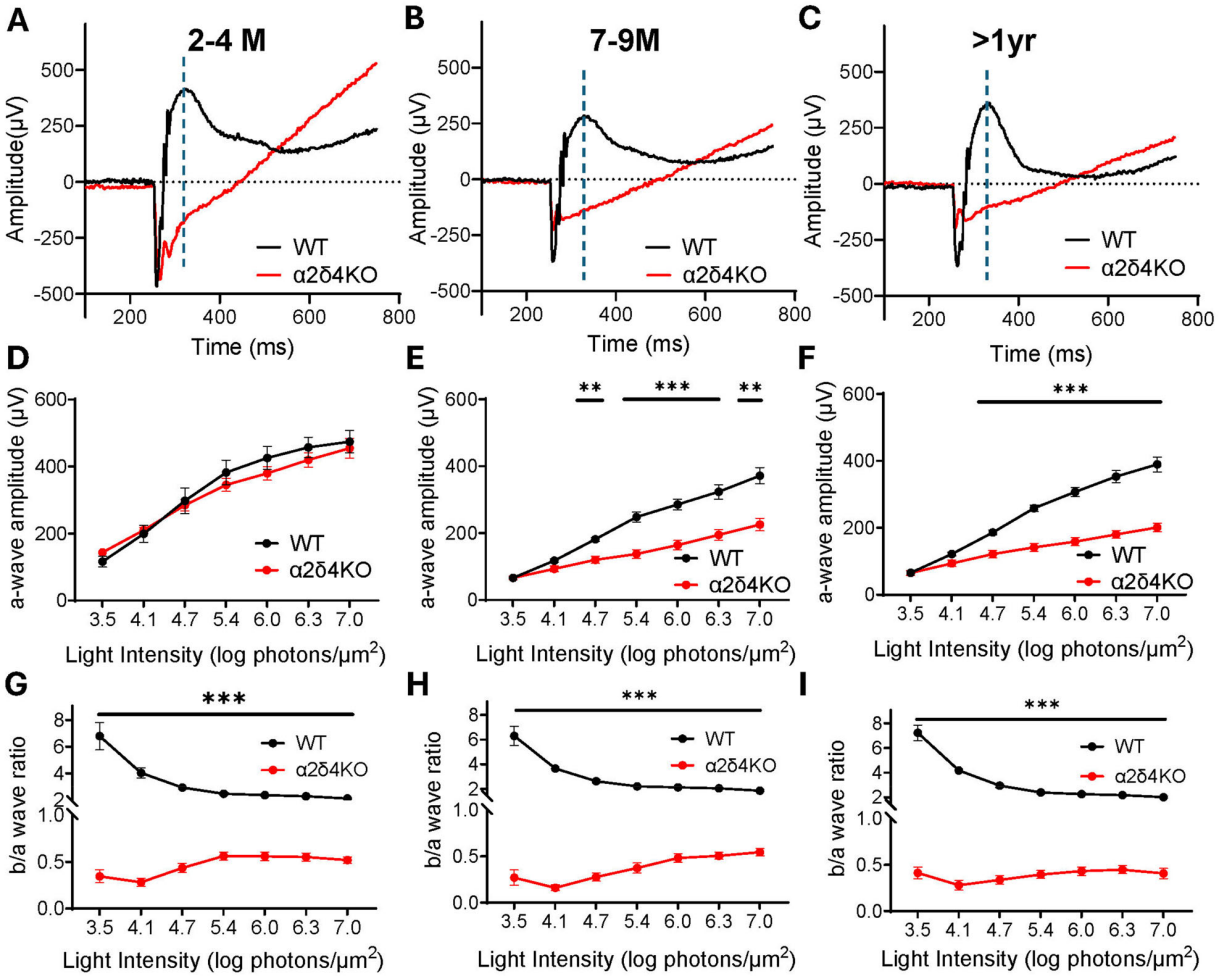
Electroretinography (ERG) study demonstrates progressive decline in photoreceptor function but unaltered synaptic transmission in α2δ4KO mice. **A**–**C** Representative traces of dark-adapted ERG response to light intensity of 6.3 log photons/µm^2^ stimulating both rods and cones from WT (*black*) and KO (*red*) mice at (**A**). 2–4 months, **B** 7–9 months, and **C** >1 year. Broken line indicates the peak of the WT b-wave, which was used as a reference for determining b-wave amplitude in KO retinas. **D**–**F** Quantification of a-wave amplitudes across increasing light intensities in WT and KO mice at (**D**). 2–4 months, **E** 7–9 months, and **F** >1 year (*n* = 7 to 11 mice per group). **G**–**I** Quantification of b/a wave ratio in WT and KO mice at (**G**). 2–4 months, **H** 7–9 months, and **I** >1 year. (*n* = 7 to 11 mice per group). Error bars are SEM, unpaired t-test. **p < 0.01, ***p < *0.001; points without an asterisk are not significant (ns)*.

To assess how retinal function is affected during degeneration, we performed ERG on α2δ4 KO and WT mice at the aforementioned ages under both dark and light adapted conditions to evaluate both rod and cone photoreceptor responses. We first examined the ERG a-waves, which represent the function of photoreceptors. For 2–4 months animals, the a-wave amplitude of both dark and light-adapted ERG remained unchanged (Fig. 2A, D**;** Supplementary Fig. 2A, D), confirming previous findings that phototransduction is not impaired by removal of α2δ4 [19, 21]. In contrast, we observed a significantly reduced a-wave in both dark and light-adapted ERG across flash intensities in both 7–9 months and >1 yr group (Fig. 2B, C, E, F; Supplementary Fig. 2B, C, E, F). Moreover, the amplitude of a-wave was not further reduced in >1 yr KO animals (Supplementary Fig. 2G). These results are consistent with our histological findings and suggest that both rod and cone photoreceptor function are compromised during the degeneration.

ERG b-waves originate from the bipolar cells and are thus used to assess synaptic function. We previously showed that α2δ4 is required for the optimal synaptic transmission for both rod and cone circuits, evidenced by severely impaired b-wave in young α2δ4 KO retinas [19]. Given the reduction of a-wave amplitudes in aged KO animals, we examined how synaptic transmission is affected during degeneration by comparing the ratio of ERG b-wave amplitude to that of a-wave (b/a ratio). We found that ERG b/a ratios of the KO animals in all age groups are drastically diminished (Fig. 2G–I). Surprisingly, the ERG b/a ratios of the aged KO animals were overall comparable to those of the young ones (Supplementary Fig. 2H, I). This analysis suggests that the impaired photoreceptor synaptic transmission efficiency remained relatively stable during the mild photoreceptor degeneration.

### Synaptic remodeling is augmented in aged α2δ4 KO retina

Synaptic remodeling has been proposed as a mechanism to maintain retinal circuit function during degeneration [6, 34]. Given the relatively unaltered synaptic transmission in aged α2δ4 KO retinas, we next studied whether and how synaptic remodeling in the α2δ4 KO retinas changes during aging since it has been previously shown that BC dendrites sprout into ONL in the young α2δ4 KO retinas [19, 21].

**Fig. 3:**
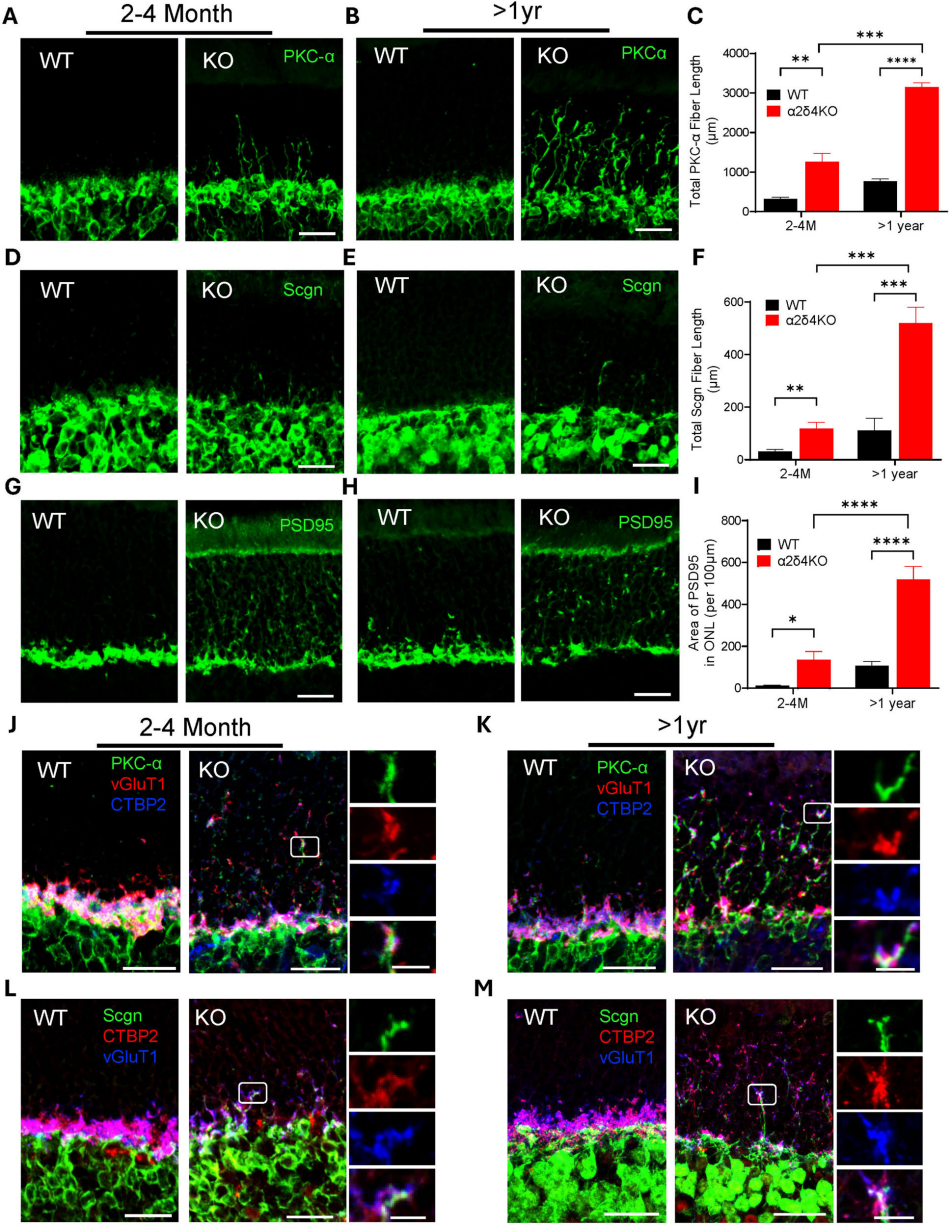
Remodeling of bipolar cell dendrites and photoreceptor terminals is augmented in aged α2δ4KO mice. **A**, **B** Confocal images of retinal sections from WT and α2δ4KO mice at (**A**). 2–4 months, and **B** >1 year stained with anti-PKC-α to label rod bipolar cells (RBCs). Scale bar, 50 μm. **C** Quantification of the total length of RBC dendritic sprouting within the ONL of WT and KO mice at 2–4 months and >1 year (*n* = 4 mice per group). **D**, **E** Confocal images of WT and KO retinal sections at (**D**). 2–4 months, and **E** >1 year stained with anti-secretagogin (Scgn) to label cone bipolar cells (CBCs). Scale bar, 50 μm. **F** Quantification of total Scgn-positive dendritic sprouting within the ONL in WT and KO mice at 2–4 months and >1 year (*n* = 4 mice per group). **G**, **H** Confocal images of WT and KO retinal sections at **G**. 2–4 months, and **H** >1 year stained with anti-PSD95 to label photoreceptor terminals. Scale bar, 50 μm. **I** Quantification of the PSD95-labeled area within the ONL of WT and KO retina at 2–4 months and >1 year (n = 4 mice per group). **J,K**. Confocal images of WT and KO retinal sections triple immunolabeled of PKC-α (green), vGlut1 (red), CTBP2 (blue) at (**J**) 2–4 months and (**K**) >1 year. Insets show higher magnification of retracted photoreceptor terminals colocalizing with sprouted RBC dendrites. Scale bar, 50 μm; inset, 5 μm. **L**, **M** Confocal images of WT and KO retinal sections triple immunolabeled of secretagogin (green), vGlut1 (blue), and CTBP2 (red) at **L** 2–4 months and (**M**) >1 year. Insets show higher magnification of retracted cone terminals colocalizing with sprouted CBC dendrites. Scale bar, 50 μm; inset, 5 μm. Error bars are SEM, unpaired t-test. *p < 0.05, **p < 0.01, ***p < 0.001, ****p < *0.0001*.

To investigate BC dendritic sprouting, we immunolabeled retina cryosections from mice of both genotypes at 2–4 months and >1 yr using PKC-α and secretagogin to label rod bipolar cells (RBCs) and cone bipolar cells (CBCs) [35], respectively. Strikingly, we found that the sprouting of RBC dendrites is significantly augmented in >1 yr group compared to the 2–4 months group, as quantified by sprouting total length and density (Fig. 3A–C; Supplementary Fig. 3A). As for CBCs, their dendrites also sprout into ONL at 2–4 months albeit at a much lower extent and frequency (Fig. 3D, F). Nevertheless, the extent of CBC dendrites remodeling significantly increased in the >1 yr animals (Fig. 3E, F; Supplementary Fig. 3B).

To check how photoreceptor axonal terminals respond, we labeled photoreceptor terminals with anti-PSD95 antibody. We found that PSD95-labeled area in the OPL of >1 yr KO retinas was significantly reduced compared to 2–4 months KO while its labeled area in ONL was significantly increased (Fig. 3G–I), suggesting that more photoreceptor terminals retracted during aging. To see whether cones terminals are retracted, we performed cone arrestin staining and found that while most cone terminals in the 2–4 months old KO retinas remained in the OPL, some retracted to the ONL in the >1 yr retinas (Supplementary Fig. 3C, D).

To see whether the remodeled neurites of photoreceptors and BCs stay connected and form ectopic synapses, we triple-labeled the retinas from both genotypes with photoreceptor terminal marker vGluT1, BC markers (PKC-α or Secretagogin), and synaptic ribbon marker, CTBP2. For RBCs, many of the sprouted dendrites in the young KO animals are in close contact with vGlut1 and CTBP2 labeled structures (Fig. 3J), and these pre-post appositions are maintained in the >1 yr KO retinas (Fig. 3K). For CBCs, we observed similar pre-post juxtapositions between sprouted dendrites, presynaptic terminals, and ribbons in both young and old KO retinas (Fig. 3L, M). We next performed TEM to examine the ultrastructure of these ectopic pre-post appositions in the ONL. Strikingly, we observed numerous electron-dense structures encircled by double membrane resembling BC dendrites in the ONL of the aged KO retinas (Supplementary Fig. 3E). Moreover, these electron-dense structures are often located right next to another larger double membrane enclosed structure resembling retracted photoreceptor terminals (Supplementary Fig. 3F), supporting that the remodeled photoreceptor terminals and BC dendrites still remain connected.

Together, our data suggest that the loss of α2δ4 synergizes with aging in causing synaptic remodeling which is coordinated between photoreceptors and bipolar cells even in the absence of α2δ4. These remodeled structures could potentially be ectopic synapses that maintain the synaptic transmission in degenerating α2δ4 KO retinas.

### α2δ4 contributes to photoreceptor dysfunction and degeneration primarily through Cav1.4

Given that α2δ4 is an essential part of Cav1.4 complex, we set out to determine the extent to which α2δ4-mediated photoreceptor dysfunction and degeneration result from Cav1.4 dysfunction. To this end, we compared the functional and structural deterioration of α2δ4 KO retinas with those of Cav1.4 KO retinas, in which the pore-forming subunit of the channel is completely absent [36]. We reasoned that if Cav1.4 dysfunction were the primary driver, the deterioration profile of α2δ4 KO retinas will be similar and slightly milder than that of Cav1.4 KO retinas since there is still residual Cav1.4 preserved at α2δ4 KO photoreceptor terminals.

**Fig. 4:**
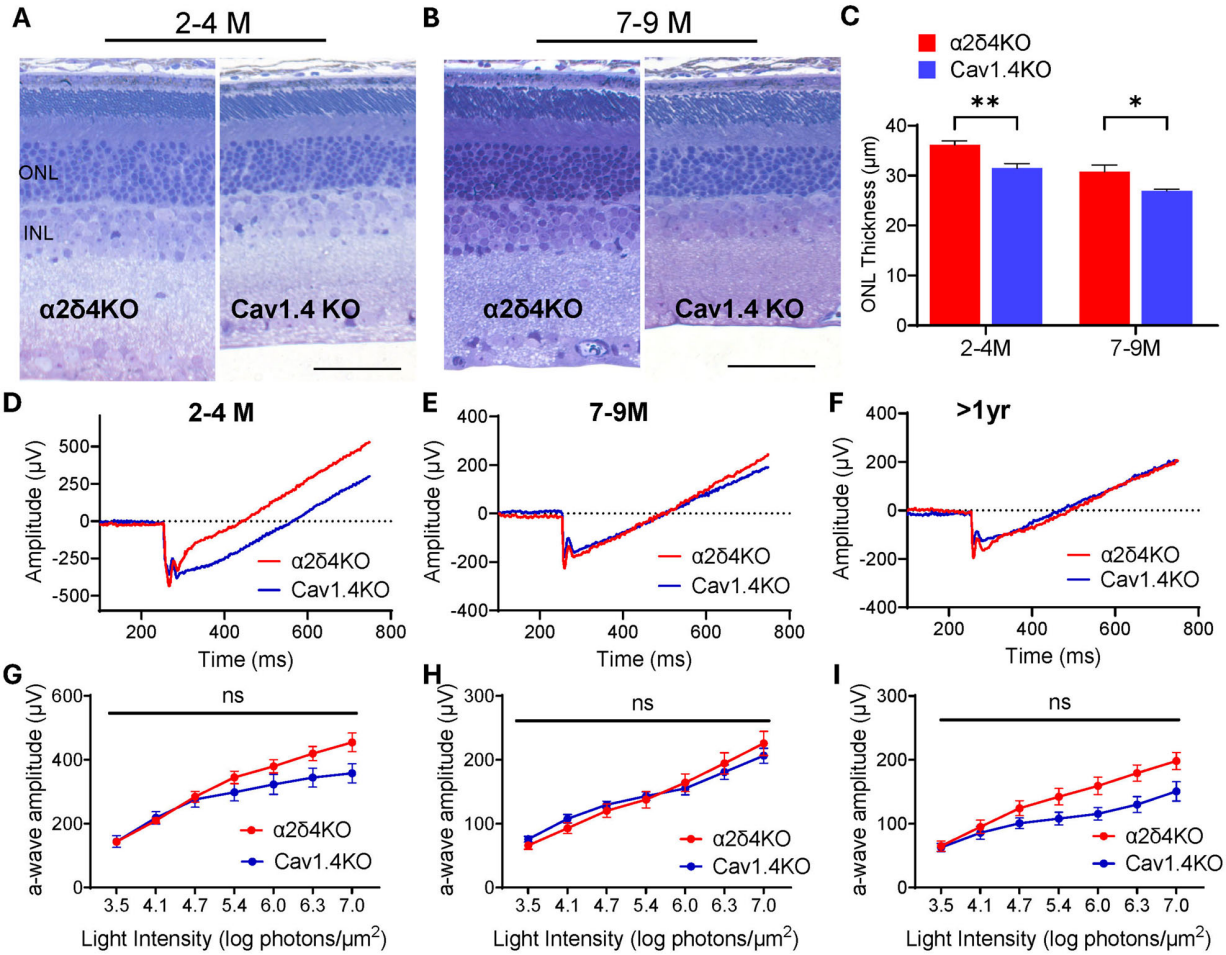
α2δ4-mediated photoreceptor dysfunction and degeneration is largely through Cav1.4. **A**, **B** Toluidine blue-stained retinal sections of α2δ4KO and Cav1.4KO mice at **A** 2–4 months and **B** 7–9 months, highlighting ONL and INL. Scale bar, 50 μm. **C** Quantification of central ONL thickness in α2δ4KO and Cav1.4KO retinas (*n* = 3 animals per group). **D**–**F** Representative dark-adapted ERG traces in response to bright flashes of 6.3 log photons/µm², stimulating both rods and cones, from α2δ4KO (*red*) and Cav1.4KO (*blue*) mice at **D** 2–4 months, **E** 7–9 months, and **F** >1 year. **G**–**I** Quantification of a-wave amplitudes across increasing light intensities in α2δ4KO and Cav1.4KO mice at **G** 2–4 months, **H** 7–9 months, and **I**. >1 year (*n* = 7–11 animals per group). Error bars, SEM; unpaired t-test. *p < 0.05, **p < *0.01, ns; not significant*.

We first compared the ONL thickness between α2δ4 KO and Cav1.4 KO retinas at 2–4 months and 7–9 months. We found that the ONL of Cav1.4 KO retina is significantly thinner than that of α2δ4 KO retina even at 2–4 months (Fig. 4A, C), and that this difference in ONL thickness persists into 7–9 months (Fig. 4B, C). The slightly more aggressive degeneration of Cav1.4 KO retina was further validated using OCT (Supplementary Fig. 4A–C). We then compared functional changes between the two KO lines by measuring dark-adapted ERG a-wave amplitude across ages. We found that the a-wave amplitudes of Cav1.4 KO retina were consistently lower than those of α2δ4 KO across flash intensities and for all ages, although the differences did not reach significance (Fig. 4D–I).

Together, our data shows that Cav1.4 KO mice share a similar yet more severe deterioration profile compared to α2δ4 KO at both functional and structural level. These findings support that disruption of Cav1.4 complex is the primary cause for α2δ4-mediated photoreceptor dysfunction and degeneration.

### Proteomic identification of TCA cycle as the top perturbed pathway in young α2δ4 KO retina

How does calcium channel deficit at the synaptic terminal trigger overall photoreceptor degeneration? To explore the underlying molecular mechanism, we carried out a proteomic study to profile protein changes in the α2δ4 KO retina at both early and late stage, aiming to uncover the early molecular and pathways changes that drive photoreceptor death.

**Fig. 5:**
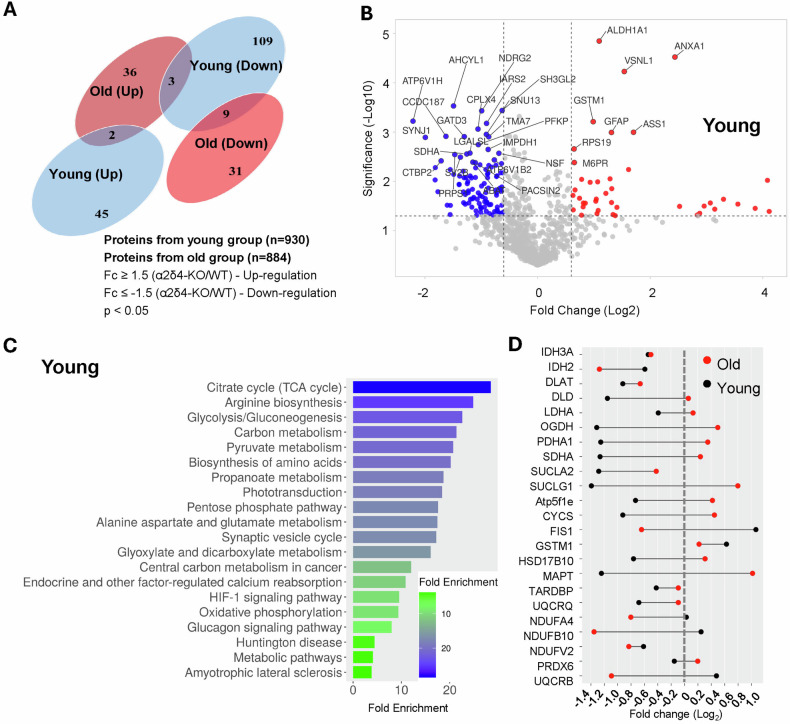
Proteomic identification of TCA cycle as the top perturbed pathway in young α2δ4KO retina. **A** Venn diagram comparing differentially expressed proteins (DEPs) in α2δ4KO retinas at 2–4 months (young) and > 1 year (old). **B** Volcano plot of DEPs in the young α2δ4KO retinas. DEPs with fold change (FC) ≥ 1.5 are highlighted in blue (downregulated) and red (upregulated). Statistical significance determined by t-test (*p* ≤ 0.05); *n* = 4 mice. **C** KEGG pathway enrichment analysis showing the top 20 pathways perturbed in young α2δ4KO retinas. **D** Dumbbell plot illustrating fold changes in expression of proteins associated with TCA cycle in young (black dots) and old retinas (red dots). Statistical significance determined by unpaired t-test (*p* ≤ 0.05).

Overall, we identified 930 and 884 differentially expressed proteins (DEPs) from the young (2–4 months) and the old (17 months) group, respectively. After applying stringent filters of both fold change and p-value, we ended up with 154 DEPs for the young group and 67 total DEPs for the old group (Fig. 5A). The few overlaps in the DEPs between young and old groups suggest that the proteomic landscapes of α2δ4 KO retinas at early and late stages are rather different.

We then ranked the DEPs by *p*-value and identified the top 40 DEPs in the KO retinas for both age groups (Fig. 5B; Supplementary Fig. 5A–C). We noticed that many of these DEPs in the young group are involved in energy metabolism while those in the old group are involved in phototransduction. This was confirmed by the pathway analysis which revealed that the top perturbed pathway in the young and old α2δ4 KO retinas turned out to be tricarboxylic acid (TCA) cycle and phototransduction, respectively (Fig. 5C; Supplementary Fig. 5D). We validated this analysis by calculating the fold changes of all the proteins involved in each pathway and found that almost all involved proteins are significantly downregulated (Fig. 5D; Supplementary Fig. 5E).

Together, our proteomic studies revealed a specific downregulation of TCA cycle in the young α2δ4 KO retinas and suggest impaired metabolism as one driving force for α2δ4-associated photoreceptor degeneration.

### Synaptic mitochondria are preferentially damaged in young α2δ4 KO retina

**Fig. 6:**
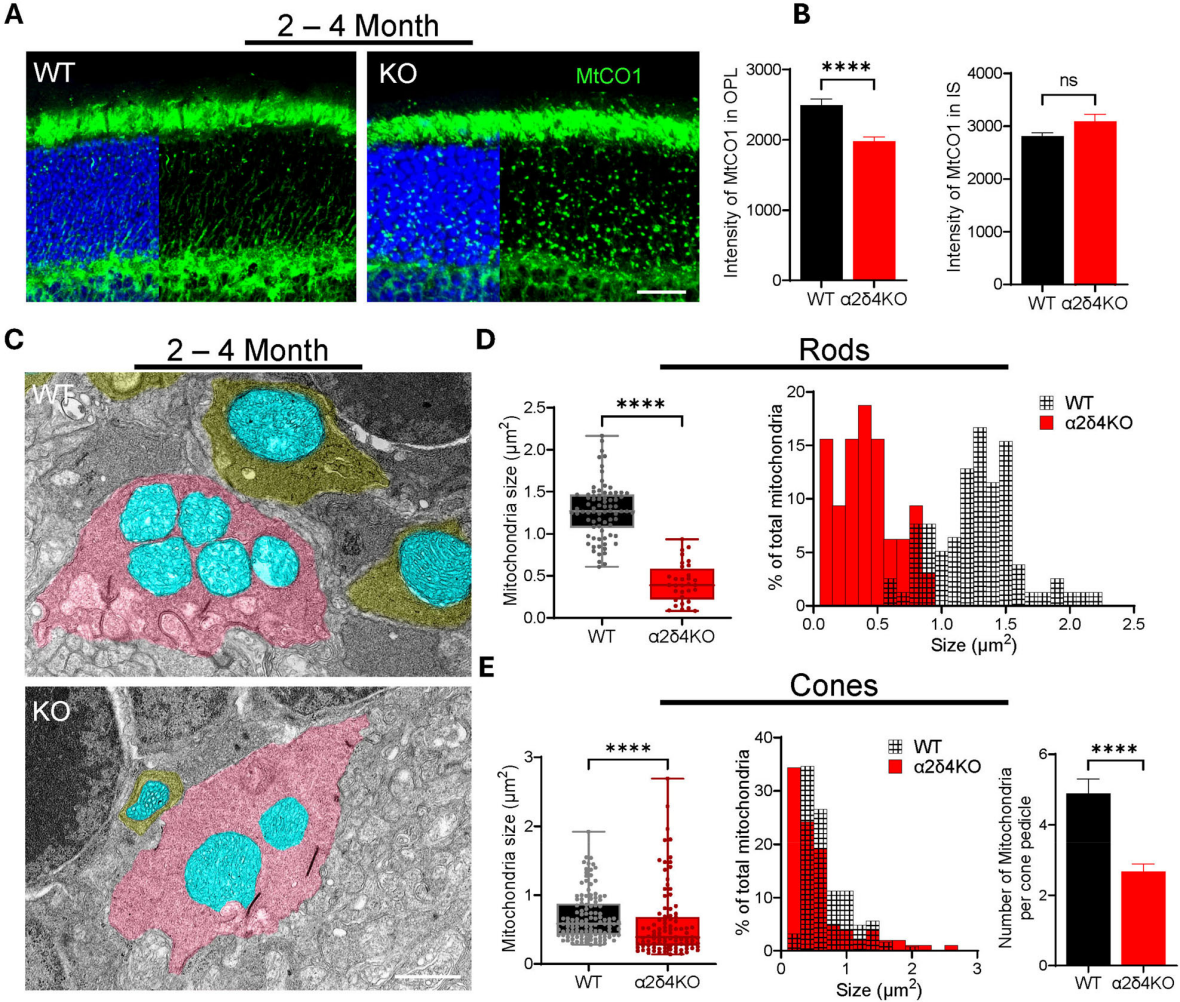
Loss of α2δ4 causes early mitochondrial damage specifically in synaptic terminals. **A** Confocal images of MtCO1-stained retinal sections of WT and KO mice at 2–4 months. Arrows indicate MtCO1 staining in the ONL. Scale bar, 50 μm. **B** Quantification of mean MtCO1 fluorescence intensity in the OPL and IS (*n* = 4). **C** Representative TEM images showing ultrastructure of rod spherules (*yellow*) and cone pedicles (*pink*) containing mitochondria (*blue*) in WT and KO 2–4 months retinas. Scale bar, 1 μm. **D** Quantification of the average size (*left*) and distribution (*right*) of mitochondria within rod spherules of WT and KO 2–4 months mice retina (n; WT: 78 rod terminals from 3 mice; KO: 32 terminals from 3 mice). **E** Quantification of the average size (*left*), distribution (*middle*) and numbers (*right*) of mitochondria within cone pedicles of WT and KO 2–4 months mice retina. (n; WT: 124 cone terminals from 3 mice; KO: 99 terminals from 3 mice). Error bars are SEM, unpaired t-test. ****p < *0.0001, ns; not significant*.

Given that mitochondria are the central hubs for cellular metabolism and calcium homeostasis [37, 38], we decided to examine whether mitochondria are affected in the α2δ4 KO retinas at 2–4 months and 7–9 months. We immunolabeled both WT and age-matched α2δ4 KO retinas against cytochrome c oxidase subunit I (MTCO1), a protein crucial for cellular energy production and used as a mitochondrial marker [39, 40]. In the WT retina, MTCO1 signal is enriched in both IS and OPL, consistent with the mitochondria location in mouse photoreceptors (Fig. 6A) [41, 42]. In the α2δ4 KO retina, we found that the signal is scattered throughout ONL, implying that this signal comes from the mislocalized mitochondria likely residing in the retracted photoreceptor terminals (Fig. 6A). We quantified the intensity of MTCO1 labeling in both the IS and OPL and found that MTCO1 level in the OPL is significantly reduced in the KO retinas of both ages (Fig. 6B; Supplementary Fig. 6A, B). Interestingly, MTCO1 level in the IS of KO retinas showed an increasing trend but did not reach significance (Fig. 6B**;** Supplementary Fig. 6A, B).

To examine how mitochondria is impaired at ultrastructural level, we performed TEM on WT and KO retinas at 2–4 months and 7–9 months. We first checked the mitochondria in the photoreceptor terminals. In the WT retina, each rod spherule contains one large mitochondrion while each cone pedicle contains multiple smaller mitochondria (Fig. 6C). In the KO retina, rod spherules are difficult to identify even at 2–4-months due to much reduced size as well as drastic terminal retraction to ONL. For the remaining rod spherules in the OPL, they still contain one mitochondrion, but with a much smaller size (Fig. 6C, D). For the cones, not only is the average size of the mitochondria significantly reduced, but also the number of mitochondria in each cone pedicle (Fig. 6C, E). We performed the same TEM analysis on the 7–9-month-old retinas and found no further changes in the size and number of mitochondria in both rod and cone photoreceptor terminals (Supplementary Fig. 6C–E). We also examined the ultrastructure of mitochondria in the inner segment (IS). In WT retinas, the IS mitochondria are mostly elongated and distributed along the IS in both 2–4 and 7–9 months groups (Supplementary Fig. 6F, G). In the KO retinas, majority of the IS mitochondria are still elongated at 2–4 months but many became shorter and relatively swollen at 7–9 months (Supplementary Fig. 6F, G).

Together, these data support our proteomic findings and demonstrate that mitochondrial damage, particularly at the synaptic terminals, is one early downstream effector of α2δ4 dysfunction which could underlie the overall photoreceptor dysfunction and death.

### Ca^2+^ crosstalk within presynaptic photoreceptor terminals is disrupted upon α2δ4 loss

How are synaptic mitochondria selectively damaged in the young α2δ4 KO photoreceptors? We reasoned that the damage is likely caused by some local events within the photoreceptor terminals. It has been shown that Ca^2+^ influx through VGCC is directly coupled to ER Ca^2+^, which is tightly connected to the mitochondria in several types of cells, including photoreceptors [43–48]. Given that Ca^2+^ influx at the terminals is much reduced in α2δ4 KO retinas [19, 21, 25], we speculated that impaired Ca^2+^ influx leads to disrupted presynaptic Ca^2+^ crosstalk and homeostasis, which triggers the observed mitochondria damage.

**Fig. 7:**
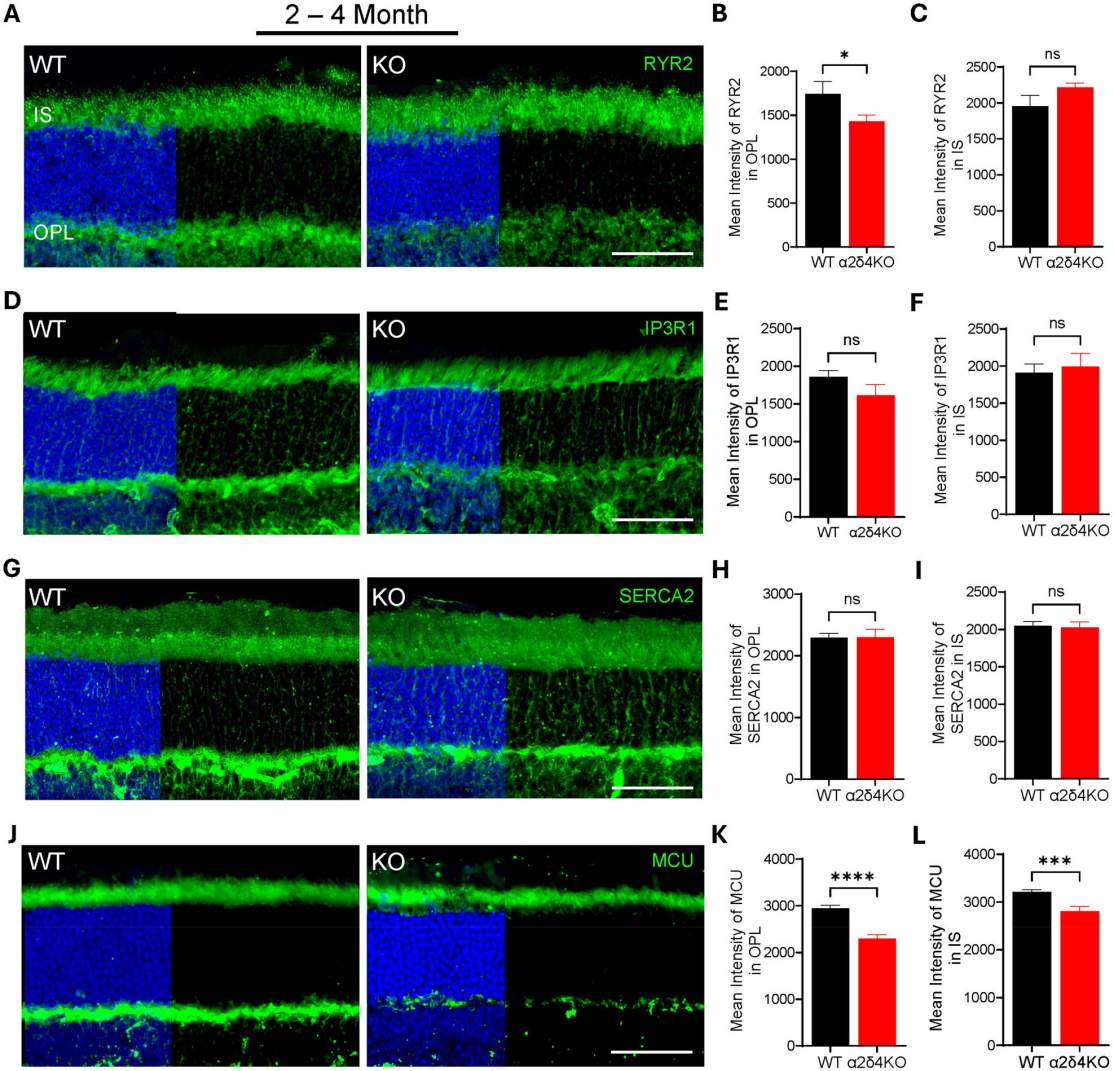
α2δ4 loss disrupts presynaptic Ca²^+^ coupling within photoreceptor terminals. **A** Confocal images of RYR2-stained retina sections of WT and KO mice at 2–4 months. Scale bar, 50 μm. **B**, **C** Quantification of RYR2 immunofluorescence intensity in the **B** OPL and **C** IS of WT and KO retinas at 2–4 months and 7–9 months (*n* = 4 mice per group). **D** Confocal images of IP3R1-stained retina sections of WT and KO mice at 2–4 months. Scale bar, 50 μm. **E**, **F** Quantification of IP3R1 immunofluorescence in the **E** OPL and **F** IS of WT and KO retina at 2–4 months and 7–9 months (*n* = 4 mice per group). **G** Confocal images of SERCA2-stained retina sections of WT and KO mice at 2–4 months. Scale bar, 50 μm. **H**, **I** Quantification of SERCA2 immunofluorescence intensity in the **H** OPL and **I** IS of WT and KO retina at 2–4 months and 7–9 months (*n* = 4 mice per group). **J** Confocal images of MCU-stained retina sections in WT and KO mice at 2–4 months. Scale bar, 50 μm. **K**, **L** Quantification of MCU immunofluorescence intensity in the **K** OPL and **L** IS of WT and KO retina at 2–4 months and 7–9 months (*n* = 4 mice per group). Error bars are SEM, unpaired t-test. *p < 0.05, ***p < 0.001, ****p < *0.0001, ns; not significant*.

To test, we first focused on two ER calcium channels, inositol 1,4,5-triphosphate receptor (IP3R), and ryanodine receptor (RYR), which mediate Ca^2+^ release from ER to cytosol [49, 50]. Immunolabeling of IP3R1 and RYR2 showed strong signal in both IS and OPL in WT retinas, consistent with previously published results (Fig. 7A, D; Supplementary Fig. 7A, C) [51–54]. The fluorescent intensity of RYR2 is significantly reduced in the 2–4-month and 7–9-month-old α2δ4 KO retina (Fig. 7A, B**;** Supplementary Fig. 7A, B), and this reduction is specific for OPL but not for IS (Fig. 7C**;** Supplementary Fig. 7C). As for IP3R1, the fluorescent intensity in the 2–4-month-old KO retina remained unchanged in both OPL and IS (Fig. 7D-F). Interestingly, the signal in the IS, but not OPL, is significantly increased in the 7–9-month-old KO retinas (Supplementary Fig. 7D-F). In addition to the ER calcium channels, we also checked the Ca^2+^ pump, sarcoplasmic/endoplasmic reticulum Ca^2+^-ATPase (SERCA2), which transports cytosolic Ca^2+^ into ER [55]. Immunolabeling of SERCA2 in the WT retina sections showed strong signals in the OPL (Fig. 7G; Supplementary Fig. 7G). Quantification of the fluorescent intensity showed no significant change in both OPL and IS of 2–4 months and 7–9-month-old KO animals (Fig. 7H–I**;** Supplementary Fig. 7H–I). The differential impact of α2δ4 loss on ER calcium channels and pump implicates a disruption of ER Ca^2+^ homeostasis, which presumably leads to ER stress [54, 56, 57]. This was supported by our quantitative western blot analysis, which showed significant upregulation and activation of both CHOP and eIF2a, two widely used ER stress markers [58, 59] in the 2–4 months α2δ4 KO retinas (Supplementary Fig. 7J–M).

The Ca^2+^ transfer from ER to mitochondria is crucial for maintaining mitochondria structure and function [50, 60]. We thus examined mitochondria calcium uniporter (MCU), which mediates Ca^2+^ influx into mitochondria [61–63]. Consistent with the mitochondria location in the mouse photoreceptors, we observed strong signal in both IS and OPL in the WT retina (Fig. 7J**;** Supplementary Fig. 7N). In the absence of α2δ4, MCU levels at both OPL and IS are significantly diminished at both 2–4 months and 7–9 months (Fig. 7J– L**;** Supplementary Fig. 7N–P), suggesting that mitochondrial Ca^2+^ homeostasis is disrupted in the α2δ4 KO photoreceptors.

Together, our data provided evidence of an impaired calcium crosstalk between synaptic plasma membrane, ER, and mitochondria, which could underlie the mitochondria damage and ER stress in α2δ4 KO retinas.

## Discussion

**Fig. 8:**
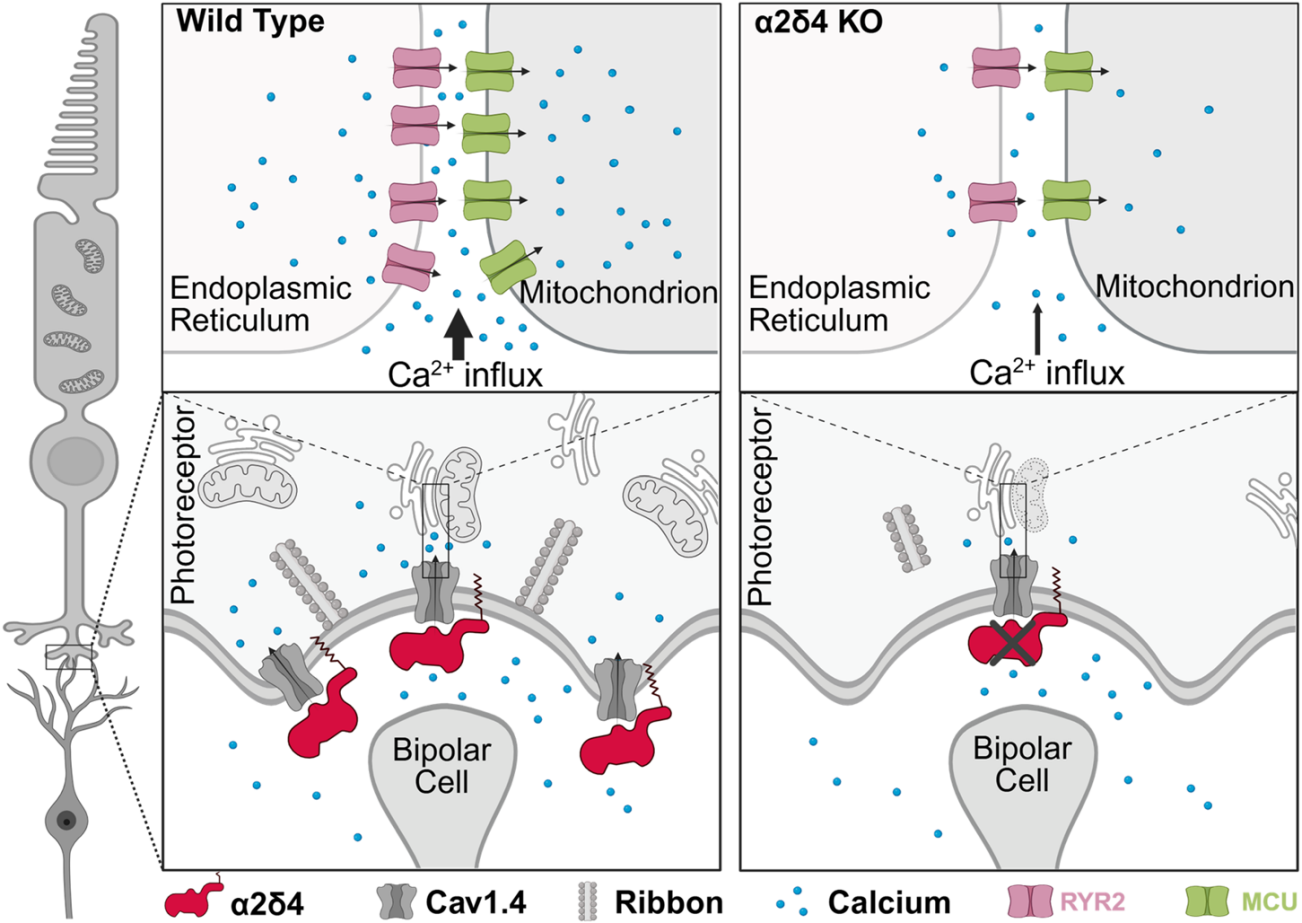
Proposed mechanism by which loss of α2δ4 leads to photoreceptor degeneration. In the wild-type (WT) retina (*left*), α2δ4 stabilizes the Cav1.4 channel at the active zone to support continuous Ca²^+^ influx required for neurotransmission and calcium dynamics within ER and mitochondria via RyR2 and MCU, which is essential for Ca²^+^ homeostasis and metabolic coupling. In the absence of α2δ4 (α2δ4KO) (*right*), Cav1.4 abundance and function are much reduced, which leads to reduced Ca²^+^ influx, and impaired ER–mitochondrial Ca²^+^ exchange. This dysregulation of synaptic calcium crosstalk may contribute to the damage of synaptic mitochondria and together contribute to the progressive photoreceptor degeneration. Created in BioRender.com.

Several synaptic genes have been implicated in retinal dystrophies [64–67], yet how local synaptic deficits lead to the overall cell death remains unclear. Mutations in CACNΑ2D4, which encodes the protein α2δ4, have been identified in patients with cone-rod dystrophy (CRD) [26, 30, 68, 69]. Here, we showed that loss of α2δ4 in mice leads to a late-onset and mild photoreceptor degeneration that closely mirrors the clinical phenotype [26, 69]. Our data indicate that α2δ4-associated degeneration primarily arises from disruption of the Cav1.4 calcium channel. Mechanistically, we discovered the TCA cycle as one of the most perturbed signaling pathways in the young α2δ4 KO retinas, prior to overt photoreceptor degeneration. This was supported by light and electron microscopy studies showing reduction of key mitochondrial markers, abnormal mitochondria distribution, reduced mitochondrial size, and number. Notably, these early changes of mitochondria are mostly observed at the photoreceptor synaptic terminals. We further provided evidence that the observed synaptic mitochondrial dysfunction is likely triggered by perturbed Ca^2+^ crosstalk among the plasma membrane, ER, and mitochondria (Fig. 8). Collectively, this study uncovers a molecular mechanism centered on synaptic mitochondria and Ca^2+^ signaling that connects synaptic dysfunction to the progressive degeneration of photoreceptors.

### α2δ4 associated photoreceptor remodeling and degeneration

The role of α2δ4 in photoreceptors degeneration was first implicated in a study where two siblings carried a homozygous single point mutation in CACNΑ2D4 gene [26]. Both patients share a slowly progressing cone dystrophy noted in the third decades of their life. In a later study, two patients carrying different homozygous mutations showed diminished ERG responses, which remained stable across 8-9 years [30]. This is consistent with the late onset and mild degeneration of photoreceptors in the α2δ4 KO mice till 15–17 months. Our findings are consistent with a previous study using a different α2δ4 KO model, which displayed a significant thinning of ONL at 6 months [21]. Together with another earlier study using a spontaneous CACNA2D4 mutant mouse [32], these animal studies substantiate the role of α2δ4 in photoreceptor degeneration and support the value of α2δ4 KO mice in studying the pathology of photoreceptor degeneration.

It has been assumed that α2δ4 associated degeneration is through the disruption of Cav1.4 given its essential role in calcium channel function [70]. However, it is possible that α2δ4-associated degeneration involves channel-independent roles of α2δ proteins in mediating extracellular molecular adhesions and signaling, as has been shown for other α2δ isoforms [70–72]. Our study demonstrated that α2δ4 KO and Cav1.4 KO photoreceptors share an overall similar degeneration profile, with α2δ4 KO photoreceptors displaying a slightly slower onset and milder degeneration. Our results are consistent with a recent study comparing all-cone models of Cav1.4 KO and α2δ4 KO [73] and support the hypothesis that the extent of calcium channel disruption correlates with the extent of photoreceptor degeneration. Notably, Maddox and colleagues have shown the non-conductive role of Cav1.4 in both rod and cone synapses presumably through scaffolding [74, 75]. This led to the question of to what extent the photoreceptor degeneration is contributed by Ca^2+^ signaling impairment versus the structural scaffolding disruption. Regus-Leidig and colleagues compared the degeneration profiles of two Cav1.4 mutant models, a loss-of-function (LOF) mutant (ΔEx14–17) and a gain-of-function (GOF) mutant (I745T). Interestingly, the authors discovered that I765T GOF mice displayed a greater ONL thinning than ΔEx14-17 LOF mice, despite that I765T retinas have a better-preserved synaptic ribbon structure [76]. This argues that disruption of Ca^2+^ influx plays a greater role in Cav1.4 associated photoreceptor degeneration than its non-conducting structural role.

One key structural change in α2δ4 KO retina, as well as retinas lacking other essential parts of the Cav1.4 complex, is the retraction of photoreceptor terminals and sprouting of BC dendrites [19, 21, 76–80]. Interestingly, similar neurite remodeling was also observed in retinas lacking LKB1, which plays an essential role in cellular energy metabolism [81, 82]. Our findings that mitochondria are damaged in α2δ4 KO retinas suggest that mitochondria may be one converging mechanism controlling photoreceptor remodeling.

We showed that the extent of remodeling is significantly increased in aged KO retinas and that many of the retracted photoreceptor terminals still maintain contact with BC dendrites. This suggests that the remodeling could be one mechanism that contributes to the unaltered synaptic transmission efficiency measured by ERG b/a ratio. This is in line with the observations made in retinal degeneration models as well as partial photoreceptor deletion models where BC dendrites remodeled to restore synaptic input from photoreceptors [83–86]. Our findings that RBCs dendrites sprout more than CBCs in the absence of α2δ4 is consistent with a previous study where photocoagulation was used to selectively destroy photoreceptors in adult rabbit retinas [87]. This study showed that RBCs extend their dendrites to form new synapses with the remaining photoreceptors while secretagogin labeled CBCs did not exhibit such dendritic remodeling. The cell type dependent remodeling of BC dendrites was even observed among subtypes of ON-CBCs in response to partial cone ablation [83, 85]. Our study using α2δ4 KO mice suggests that the cell-type dependent remodeling of BC dendrites is primarily driven by the distinct intrinsic mechanism of BCs rather than by circuit level mechanism since the synaptic communication between photoreceptors and BCs is essentially ablated in the absence of α2δ4.

### Metabolism and mitochondria in photoreceptor degeneration

One of the key findings of this study is that mitochondria located in the photoreceptor terminals are drastically reduced in size and number in the absence of α2δ4 long before photoreceptor degeneration was observed. Mitochondrial size and number are directly correlated to energetic output and cell survival [88–90]. Moreover, the early onset of mitochondria damage coincides with the occurrence of photoreceptor apoptosis, suggesting damage of the synaptic mitochondria as one driving force in α2δ4-associated photoreceptor degeneration.

Early mitochondrial deficits have been observed in several other mouse models of photoreceptor degeneration, including retinitis pigmentosa (RP), chronic HIFs (hypoxia-induced factors) activation and light-induced degeneration [91–94]. This observation is further substantiated by a recent study showing a common early defect in autophagy and mitochondria in several genetically and functionally distinct IRD mouse models [95].

Unlike the models mentioned above, the α2δ4 KO mice display a much milder degeneration profile, which is proceeded by local deficits only at the synaptic terminals. Yet the photoreceptors in α2δ4 KO retina share similar deficits in energy metabolism and mitochondrial function at an early stage. Our study thus corroborates and extends previous findings by pointing to mitochondrial deficits as a unifying mechanism of early photoreceptor degeneration independent of perturbation type and origin. Whether various types of perturbations trigger mitochondrial damage to a similar extent and through a common pathway remains incompletely understood and will be an exciting future research direction. Nevertheless, targeting mitochondria using mitochondria-protective agents or metabolic boosters is emerging as a promising therapeutic strategy for neuroprotection [92, 96–100]. It will be interesting to test whether boosting mitochondria function can slow down the degeneration in α2δ4 KO retinas.

In addition to the damage of mitochondria in the OPL, we also observed significant translocation of mitochondria into the ONL. Mislocalization of damaged mitochondria has been reported in cone photoreceptors of zebrafish [101]. Notably, the mislocalized mitochondria were observed both inside and outside of the photoreceptors, which were mostly taken up by Müller cells. We speculate that Müller cells are likely to play a role in clearing the ONL mitochondria in the α2δ4 KO retinas, given that Müller cell activation has been reported in Cav1.4 mutant mice [102]. Since mitochondria release from photoreceptors have been observed in other species, including mice and primates [103, 104], it seems a conserved mechanism that Müller cells serve as a critical clearance mechanism for damaged mitochondria produced in photoreceptors.

### Presynaptic Ca^2+^ signaling in photoreceptor degeneration

Ca^2+^ influx through VGCCs on the plasma membrane is coupled with ER Ca^2+^ through calcium induced calcium release (CICR), which has been shown as a critical process for synaptic function [45, 47]. We and others have previously shown that loss of α2δ4 leads to significant reduction of Ca^2+^ influx at both rods and cones terminals [19, 21, 25]. Here, we showed that ER calcium channel, RYR2, is significantly reduced in the photoreceptor terminals. Increased expression of RYR2 has been reported in a mouse model of photoreceptor degeneration where CNG channel is deleted [105]. Notably, deletion of RYR2 specifically in the cones improves localization of phototransduction proteins in the CNG KO retina and reduces ER stress and cone apoptosis [106]. It remains to be studied as to whether reduced RYR2 in α2δ4 KO mice is maladaptation or compensation for the disrupted VGCC function.

The potential change in ER Ca^2+^ is expected to disrupt mitochondria Ca^2+^ homeostasis through ER-mitochondria Ca^2+^ transfer mediated by MCU, which is activated by local high Ca^2+^ concentration at the ER–mitochondria contact sites (ERMCSs) [88, 107–109]. It has been shown that MCU overexpression in zebrafish cone photoreceptors leads to mitochondria Ca^2+^ increase as well as mitochondria damages [110]. The reduced MCU in α2δ4 KO retina indicates disrupted mitochondria Ca^2+^ homeostasis and suggests that the observed structural deficits of mitochondria are likely caused by impaired mitochondrial Ca^2+^. Whether and how mitochondrial Ca^2+^ is changed in α2δ4 KO photoreceptors remains to be experimentally tested to further establish the causal relationship between disrupted Ca^2+^ and mitochondrial structure and function.

The hypothesis that local Ca^2+^ signaling within the presynaptic terminals contributes to mitochondrial damage in α2δ4 KO retina is further supported by the observation that synaptic mitochondria are severely damaged while those in the inner segments remained relatively unchanged. Interestingly, this functional heterogeneity of photoreceptor mitochondria is also observed in an EAE (Experimental Autoimmune Encephalomyelitis) mouse model of Multiple Sclerosis in which mitochondria in photoreceptor terminals are strongly compromised whereas those in photoreceptor inner segments are unaffected [111]. Notably, EAE mice also display early synaptic deficits, suggesting that the specific vulnerability of the synaptic mitochondria is due to local molecular events such as synaptic Ca^2+^ signaling.

The functional heterogeneity among different populations of photoreceptor mitochondria could explain both the late onset and slow progression of the degeneration in α2δ4 KO retina. The late onset could be contributed by the partial compensation from the unaffected IS mitochondria at early stage. Regarding the slow progression, particularly after 7–9 months, we speculate that there might be additional compensation mechanisms which kicked in around 7–9 months in the KO retinas to further combat the degeneration. One possible mechanism could be the upregulation of IP3R1 in the IS of KO retina at 7–9 months as altered IP3R expression has been shown as protective in early hypoxic damage and cone degeneration models [54, 112]. Another possible and non-mutually exclusive mechanism is the metabolic reprogramming of photoreceptors, which has been shown in other photoreceptor degeneration models [113, 114]. This speculation is supported by our proteomic results showing that many TCA related proteins are more downregulated in young KO retinas compared to aged groups. How the two populations of photoreceptor mitochondria are functionally coupled remains an interesting question. The differential response of the two populations of mitochondria in the α2δ4 KO photoreceptors make it an attractive model to study mechanisms underlying mitochondrial heterogeneity.

We believe that the proposed mechanism likely applies to photoreceptor degeneration associated with other synaptic genes such as RIMs and Bassoon [115–117]. It has been suggested that the axonal remodeling and degeneration of photoreceptors in these cases are due to disruption in synaptic transmission. However, mouse models where synaptic transmission is abolished (e.g., ELFN KO) do not exhibit obvious photoreceptor remodeling and degeneration [118, 119], arguing against that synaptic transmission per se is the underlying mechanism for remodeling and degeneration. Moreover, RIMs and Bassoon are associated with synaptic ribbons, and loss of Bassoon and RIMs has been shown to cause reduced synaptic targeting of Cav1.4 [80, 116, 117, 120]. It is thus reasonable to propose that impaired Ca^2+^ signaling and damaged synaptic mitochondria is a common mechanism underlying synaptic gene-associated photoreceptor degeneration.

## Materials and methods

### Antibodies

The following commercial antibodies were used: mouse anti-PKCα (AB11723, Abcam), rabbit anti-PKCα (P4334, Sigma-Aldrich), rabbit anti-secretagogin (RD181120100, BioVendor), sheep anti-secretagogin (RD184120100, BioVendor), rabbit anti-PSD95 (3450, Cell Signaling Technology), mouse anti-PSD95 (MA1-045, Invitrogen), guinea pig anti-vGlut1 (AB5905, Millipore), mouse anti-CTBP2 (612044, BD Biosciences), rabbit anti-cone arrestin (AB15282, Millipore), mouse anti-MtCo1 (AB14705, Abcam), rabbit anti-RYR-2 (A34455, antibodies.com), rabbit anti-IP3R1 (07-1213, Millipore), mouse anti-SERCA2 (MA3-910, Invitrogen), mouse anti-MCU (AMAB91189, Sigma Aldrich), mouse anti-CHOP (2895, Cell Signaling Technology), rabbit anti-phospho-elf2a (3398, Cell Signaling Technology), and rabbit anti-elf2a (5324, Cell Signaling Technology).

### Animals

α2δ4 knockout (Wang et al., 2017) and Cav1.4 knockout (Specht et al., 2009) mice have been described previously. Age-matched C57Bl/6 J wild-type (WT) mice were used as controls for homozygous α2δ4 knockout (KO) mice in all experiments. Both male and female mice were used in all experiments. Animals were housed under a controlled 12-hour light/12-hour dark cycle. Standard rodent chow and water were provided *ad libitum*, and mice were maintained in plastic cages with conventional bedding and enrichment. All animal procedures were conducted in accordance with National Institutes of Health guidelines and were approved by the University of Alabama at Birmingham Institutional Animal Care and Use Committee (IACUC).

### Immunohistochemistry

Eyes were enucleated, fixed in 4% paraformaldehyde for 15 min at room temperature, and cryoprotected overnight in 30% sucrose in PBS at 4 °C. Tissues were embedded in OCT compound (Fisher HealthCare, Fisher Scientific) and cryosectioned at 14 µm thickness. Sections were rehydrated in PBS, encircled with a hydrophobic barrier (PAP pen), and blocked for 1 h at room temperature in PBS containing 0.1% Triton X-100 and 10% donkey serum. Primary antibodies were diluted in PBS containing 0.1% Triton X-100 and 2% donkey serum and applied for 1 h at room temperature. Slides were washed 3–4 times for 5 min each in PBS with 0.1% Triton X-100.

Fluorophore-conjugated secondary antibodies (1:500 in PBS with 0.1% Triton X-100 and 2% donkey serum) were applied for 30 min at room temperature in the dark. Nuclei were counterstained with DAPI (DAPI Flouromount-G, SouthernBiotech) and coverslipped. Slides were cured overnight at room temperature and sealed with clear nail polish before storage at 4 °C. The antibodies used and their dilutions include: mouse anti-PKCα, 1: 250, rabbit anti-PKCα, 1:50,000, rabbit anti-secretagogin, 1:100, sheep anti-secretagogin, 1:50, rabbit anti-PSD95, 1:100, mouse anti-PSD95, 1:500, guinea pig anti-vGlut1, 1:5000, mouse anti-CTBP2, 1:1000, rabbit anti-cone arrestin, 1:250, mouse anti-MtCo1, 1:2000, rabbit anti-RYR-2, 1:100, rabbit anti-IP3R1, 1:100, mouse anti-SERCA2, 1:250, and mouse anti-MCU, 1:100.

Apoptotic cells were detected using the TUNEL Andy Fluor^TM^ 647 Apoptosis Detection Kit (ABP Biosciences, Cat. A052). Frozen retinal sections were permeabilized with Triton X-100 and incubated with the TdT reaction cocktail containing biotin-dUTP to label DNA strand breaks. Incorporated nucleotides were detected with Andy Fluor^TM^ 647-streptavidin. DNase I-treated samples served as positive controls, and TdT-omitted sections served as negative controls. Sections were mounted in antifade medium with DAPI (VECTASHIELD HardSet, Vector Laboratories).

### Confocal microscopy and image analysis

Images were acquired on a Nikon AX-R laser confocal microscope using 10×, 40×, and 60× objectives. The same acquisition settings were used for all experimental and control groups. Unless otherwise stated, images were collected from the central retina (about 100 μm from the optic nerve head), with one image each from the temporal and nasal retina per section per mouse. Z-stacks were taken at 0.5 μm intervals, and maximum intensity projections were generated using NIS Elements and FIJI (ImageJ).

For TUNEL assays, TUNEL-positive nuclei were counted across the full retinal section. The length of rod and cone bipolar cell dendritic sprouting into the ONL was measured with a custom ImageJ macro that applied Gaussian blur, thresholding, mask conversion, skeletonization, and ridge detection to trace dendrites and automatically measure total sprouting length. Sprouting density was calculated as the area of sprouting in the ONL divided by the ONL area and expressed as a percentage. Sprouting was defined as dendritic tips that extended beyond at least one ONL nucleus.

For PSD95, MtCO1, RYR2, IP3R1, SERCA2, and MCU, fluorescence intensity and area were measured by generating binary masks of the ONL, OPL, and IS in ImageJ, converting them into ROIs, and overlaying them on the original images. To account for OPL thinning and reduced photoreceptor terminal size in KO retinas (Wang et al., 2017), OPL masks were generated using PSD95 and vGlut1 to define accurate boundaries for quantification. Genotype information was not considered until after image analysis, although experimenters were not blinded during image collection.

### Electroretinography (ERG)

Mice were dark-adapted overnight before recordings and anesthetized with ketamine/xylazine (80–100 mg/kg and 8–10 mg/kg, i.p.). Corneal anesthesia was achieved with topical 0.5% proparacaine, and pupils were dilated with 1% tropicamide and 2.5% phenylephrine. To maintain corneal hydration and facilitate electrode placement, a drop of 2.5% Hypromellose (Gonak, Akorn) was applied. ERGs were recorded from the left eye using a corneal contact lens electrode, with a gold wire loop electrode on the contralateral eye serving as the reference. Mice were placed on a temperature-controlled platform inside a Faraday cage, and light stimuli were delivered via a fiber optic positioned ~1 cm from the corneal surface.

Dark-adapted (scotopic) ERGs were recorded in response to flash stimuli ranging from 0.622 log photons/µm² to 6.955 log photons/µm², followed by light-adapted (photopic) recordings after 5 min of rod-saturating background illumination. ERG signals were amplified, digitized, and acquired with a custom-built two-channel ERG system equipped with fiber optics to deliver flashes from either a 100-Watt halogen bulb or a high intensity LED source. A-wave amplitudes were measured from baseline to the trough, while b-wave amplitudes were measured from the trough of the a-wave to the subsequent peak. Implicit times were determined for both responses. Because KO traces lacked the characteristic b-wave peak, the implicit time of WT b-waves was used as a reference to determine b-wave amplitudes in KO retinas. All responses were recorded and averaged using LabView (version 6.4.1, National Instruments), then exported into Microsoft Excel and analyzed in GraphPad Prism.

### Transmission electron and light microscopy

Retinas were fixed immediately after dissection in 2% paraformaldehyde, 2.5% glutaraldehyde, and 2 mM CaCl₂ in 100 mM cacodylate buffer (pH 7.4) and incubated overnight at 4 °C. The following day, tissues were washed in cacodylate buffer with 2 mM CaCl₂, post-fixed in 2% osmium tetroxide for 1 h, and stained en bloc with 1% aqueous uranyl acetate. Samples were dehydrated through a graded ethanol and acetone series, infiltrated with resin (Spurr’s or EMBED 812), and embedded before polymerization at 60 °C for 48 h. Ultrathin sections (~70 nm) were cut with a diamond knife, mounted on copper or nickel grids, and post-stained with 3% uranyl acetate in ethanol followed by Reynold’s lead citrate.

Images were acquired on a JEOL 1400 FLASH TEM (120 kV) equipped with an AMT NanoSprint43 Mark II camera. For orientation, 0.5 µm semithin sections were cut, stained with toluidine blue, and used to guide thin sectioning. TEM images of the OPL were captured at random central retinal locations at 4000× magnification, and images of the photoreceptor inner segments (IS) were acquired at 2500×. Rod and cone terminals and their mitochondria were manually traced in ImageJ to determine terminal size, mitochondrial size, and mitochondrial occupancy (mitochondrial area/terminal area). A total of 40 images from WT retinas (*n* = 3 animals) and 70 images from KO retinas (*n* = 3 animals) were analyzed to compensate for reduced terminal density in KO mice.

For ONL and INL thickness, toluidine blue-stained semithin sections were imaged using a Zeiss Axioplan 2 microscope with a CCD camera. Two images per section were acquired, with two sections per mouse and three mice per genotype. Images were captured 100 µm from the optic nerve. Thickness was measured in ImageJ by drawing a line across the nuclear layers, with three measurements taken per image using a grid overlay for randomization. Data was analyzed in GraphPad Prism.

### Spectral domain optical coherence tomography (SD-OCT)

In vivo retinal imaging was performed using a Bioptigen Envisu R2210 SD-OCT system (Bioptigen/Leica, Durham, NC), as previously described with modifications [DeRamus et al. 121]. Mice were anesthetized with ketamine/xylazine (80 mg/kg and 8 mg/kg, i.p.), and pupils were dilated with a mixture of 1% tropicamide and 2.5% phenylephrine. Corneal anesthesia was achieved with topical 0.5% proparacaine, and lubricating drops (Refresh Relieva PF, Allergan) were applied throughout the procedure to maintain corneal hydration. Animals were positioned in a custom imaging mount to ensure stability and consistent alignment.

For each eye, a standardized rectangular volume scan protocol (1.6 mm × 1.6 mm, 1000 A-scans × 7 B-scans × 49 frames) was centered on the optic nerve head. OCT datasets were processed using InVivoVue® software (version 3.4.4, Bioptigen/Leica) and Diver® analysis modules. Images were optimized for contrast and brightness, and manual caliper measurements were used to measure retinal layer thicknesses. Output data were exported to Microsoft Excel and further analyzed in GraphPad Prism (GraphPad Software, San Diego, CA).

### Western blot

Retinal samples were homogenized by sonication in ice-cold PBS containing 150 mM NaCl, 1% Triton X-100, Complete Protease Inhibitor tablets, and Phosphatase Inhibitor Cocktail tablets (Roche). Lysates were incubated on a rocker for 30 min at 4 °C and clarified by centrifugation at 14,000 rpm for 30 min at 4 °C. Equal amounts of total protein (6 µg) were resolved on 4–20% SDS-PAGE gels (Bio-Rad, Cat. #4568096) and transferred to PVDF membranes (Bio-Rad, Cat. #1620177). Membranes were blocked for 1 h in TBST (0.1% Tween-20 in Tris-buffered saline) containing 5% skim milk, then incubated overnight at 4 °C with primary antibodies diluted in TBST containing 1% skim milk. After washing four times in TBST (10 min each), membranes were probed with horseradish peroxidase–conjugated secondary antibodies and visualized by chemiluminescence. The following primary antibodies (Cell Signaling Technology) were used: mouse anti-CHOP (1:1000), rabbit anti-eIF2α (1:1000), and rabbit anti-phospho-eIF2α (Ser51; 1:1000). Signals were captured on film and scanned using the KwikQuant Pro detection system.

The ImageJ software (Version: v1.54 g) was used to quantify the protein levels. Output data were exported to be analyzed in GraphPad Prism (GraphPad Software, San Diego, CA). Data are presented as mean ± SEM. Statistical significance determined by an unpaired t-test (*p* ≤ 0.05); *n* = 4 mice.

### Mass spectrometry

Sample preparation: Mice retinas were lysed by sonication in ice-cold PBS containing 150 mM NaCl and Complete Protease Inhibitor tablets (Roche). Cytosolic proteins were collected by ultracentrifugation at 100,000 × *g* for 30 min at 4 °C. Pellets were resuspended in ice-cold PBS buffer (150 mM NaCl, 1% Triton X-100, Complete Protease Inhibitor), sonicated, and incubated at 4 °C for 15 min. Membrane proteins were isolated by ultracentrifugation at 100,000 × *g* for 30 min at 4 °C.

Mass Spectrometry: Peptide digests (8 µL each) were injected onto a 1260 Infinity nHPLC stack (Agilent Technologies) and separated using a 75-micron I.D. x 15 cm pulled tip C-18 column (Jupiter C-18 300 Å, 5 micron, Phenomenex). This system ran in-line with a Thermo Q Exactive HFx mass spectrometer, equipped with a Nanospray FlexTM ion source (Thermo Fisher Scientific), and all data were collected in CID mode. The nHPLC was configured with binary mobile phases that included solvent A (0.1%FA in ddH2O), and solvent B (0.1%FA in 15% ddH_2_O/85% ACN), programmed as follows; 10 min at 5% B (2 µL/min, load), 90 min at 5–40% B (linear: 0.5 nL/min, analyze), 5 min at 70% B (2 µL/min, wash), 10 min at 0% B (2 µL/min, equilibrate). Following each parent ion scan (300–1200 m/z at 60k resolution), fragmentation data (MS2) was collected on the topmost intense 10 ions at 7.5 K resolution. For data dependent scans, charge state screening and dynamic exclusion were enabled with a repeat count of 2, repeat duration of 30 s, and exclusion duration of 90 s.

MS Data Conversion and Searches: The XCalibur RAW files were collected in profile mode, centroided and converted to MzXML using ReAdW v. 3.5.1. The mgf files were then created using MzXML2Search (included in TPP v. 3.5) for all scans. The data was then searched using MASCOT (Matrix Science), which was set for three maximum missed cleavages, a precursor mass window of 20ppm, trypsin digestion, variable modification C at 57.0293, and M at 15.9949 as a base setting. Searches were performed with a species-specific subset of the UniProtKB database.

Peptide Filtering, Grouping, and Quantification: The list of peptide IDs generated based on MASCOT search results was filtered using Scaffold (Protein Sciences, Portland, Oregon). Scaffold filters and groups all peptides to generate and retain only high confidence IDs while also generating normalized spectral counts (N-SC’s) across all samples for the purpose of relative quantification. The filter cut-off values were set with minimum peptide length of >5 AA’s, with no MH + 1 charge states, with peptide probabilities of >80% C.I., and with the number of peptides per protein ≥2. The protein probabilities were set to *a* >99.0% C.I., and an FDR < 1.0%. Scaffold incorporates the two most common methods for statistical validation of large proteome datasets, the false discovery rate (FDR) and protein probability. Relative quantification across experiments was performed via spectral counting, and when relevant, spectral count abundances were normalized between samples (Hyde).

### Quantification and statistical analysis

Student’s t-test and two-way ANOVA were used for all pairwise and multi-factor comparisons, as indicated in the figure legends. Sample sizes were selected to detect biologically meaningful genotype-dependent effects based on effect sizes observed in prior studies of retinal structure, synaptic organization, and function using similar approaches, including ERG, OCT, immunohistochemistry, confocal microscopy, proteomics, and electron microscopy. Independent biological replicates from multiple animals per genotype were used for all analyses, with sample sizes ranging from *n* = 3 to 4 for ultrastructural and biochemical assays to *n* = 4–11 for ERG, OCT, and immunohistochemical analyses. Exact sample sizes are reported in the figure legends.

Data from all animals and samples were included, and no data points were excluded from analysis. Animals were assigned to experimental groups based on genotype; no additional randomization was applied. Experimenters were not blinded during data acquisition; however, genotype information was not considered until the final stage of data analysis (comparison between genotypes). Assumptions of normality and variance were assessed prior to statistical testing. Data are presented as mean ± SEM. Significance is indicated by *p ≤ 0.05, **p ≤ 0.01, ***p ≤ 0.001, and ****p ≤ 0.0001. Statistical analyses and graphing were performed using GraphPad Prism.

For the proteomic data generated, two separate non-parametric statistical analyses were performed between each pair-wise comparison. These non-parametric analyses include 1) the calculation of weight values by significance analysis of microarray (SAM; cut off >|0.8| combined with 2) T-Test (single tail, unequal variance, cut off of *p* < 0.05), which were then sorted according to the highest statistical relevance in each comparison. For SAM (Golub and Xu), whereby the weight value (W) is a statistically derived function that approaches significance as the distance between the means (μ1–μ2) for each group increases, and the SD (δ1–δ2) decreases using the formula, *W* = (μ1 − μ2)/(δ1 − δ2). For protein abundance ratios determined with N-SC’s, we set a 1.5–2.0 fold change as the threshold for significance, determined empirically by analyzing the inner-quartile data from the control experiments using ln-ln plots, where the Pierson’s correlation coefficient (R) is 0.98, and >99% of the normalized intensities fell between the set fold change. In each case, all three tests (SAM, T-test, or fold change) had to pass in order to be considered significant.

## Supplementary information

Dysfunction of α2δ4 leads to photoreceptor degeneration through disrupted synaptic mitochondria and calcium crosstalk

Related Manuscript File

## Data Availability

All data generated or analyzed during this study are included in this published article and its supplementary information files.
