# Supplementary material for: Dysfunction of α2δ4 leads to photoreceptor degeneration through disrupted synaptic mitochondria and calcium crosstalk
Source: Cell Death Dis. 2026 Mar 23;17(1):337. doi: 10.1038/s41419-026-08587-3 (PMC13039829; doi:10.1038/s41419-026-08587-3)
Supplement: Supplementary file 1 — Dysfunction of α2δ4 leads to photoreceptor degeneration through disrupted synaptic mitochondria and calcium crosstalk [file 41419_2026_8587_MOESM1_ESM.pdf]

SUPPLEMENTAL MATERIALS

Dysfunction of α2δ4 leads to photoreceptor degeneration through disrupted synaptic mitochondria and calcium crosstalk

Choice I. Amieghemen[1], Trong Thuan Ung[1], Gillian N. Huskin[1], James A. Mobely[2], Melissa F. Chimento[3], Mai Nyugen[1], James Fortenberry[1], Timothy W. Kraft[1], Steven J. Pittler[1], Yuchen Wang[1*]

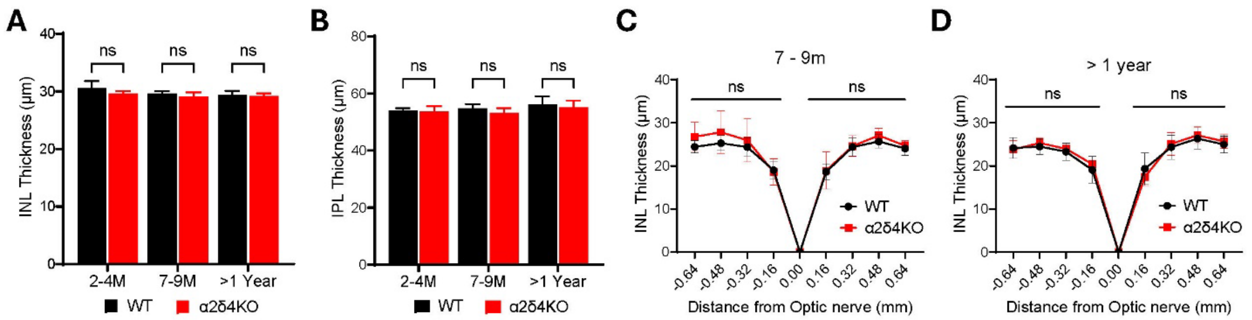

Supplementary Figure 1 (related to Figure 1). Inner nuclear and plexiform layer thicknesses remain unchanged in α2δ4KO retina.

A. Comparison of central retinal inner nuclear layer (INL) thickness using toluidine blue-stained sections from WT and KO mice at 2 – 4 months, 7 – 9 months, and >1 year (n = 3 mice per group).

B. Comparison of central retinal inner plexiform layer (IPL) thickness using toluidine blue-stained sections from WT and KO mice at 2 – 4 months, 7 – 9 months, and >1 year (n = 3 mice per group).

C – D. Quantification of INL thickness across retinal eccentricities in **C.** 7 – 9 month and **D.** >1 year WT and KO mice (n = 8 mice per group).

Error bars are SEM, unpaired t-test. *ns; not significant*

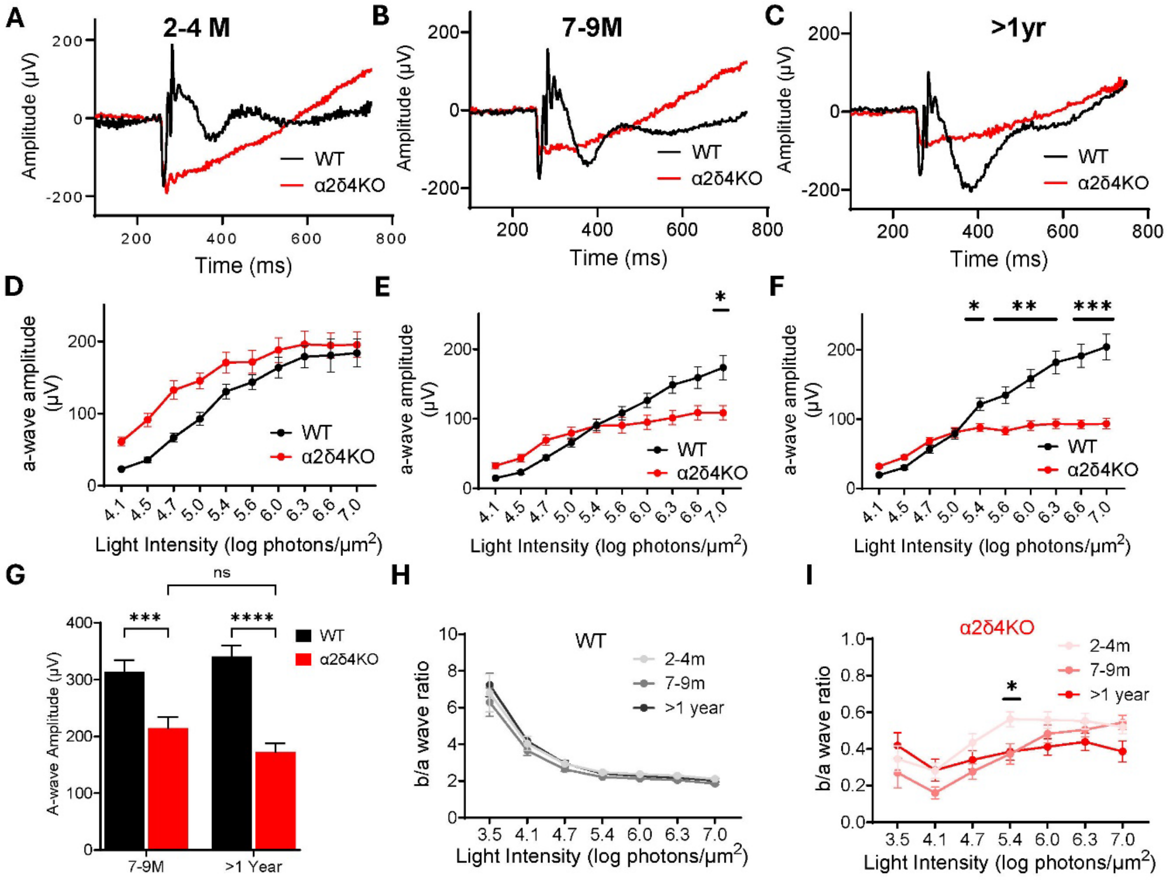

Supplementary Figure 2 (related to Figure 2): Light-adapted ERG studies reveal progressive dysfunction in α2δ4KO mice.

A – C. Representative ERG traces in response to 6.3 log photons/μm^2 with rod saturating background illumination in WT (*black*) and KO (*red*) mice at **A.** 2 – 4 months, **B.** 7 – 9 months, and **C.** > 1 year.

D – F. Quantification of a-wave amplitudes across increasing light intensities in WT and KO mice at **D.** 2 – 4 months, **E.** 7 – 9 months, and **F.** > 1 year (n = 7 to 11 mice per group).

G. Comparison of a-wave amplitude stimulated by 6.3 log photons/μm^2 stimulating both rods and cones in WT and KO mice at 7 – 9 months and >1 year.

H - I. Quantification of dark-adapted ERG b/a wave ratio in **H.** WT and **I.** KO mice at 2 – 4 months, 7 – 9 months, and > 1 year. (n = 7 to 11 mice per group).

Error bars are SEM, unpaired t-test for **D – G**, and two-way ANOVA for **H – I**. **p < 0.05, **p < 0.01, ***p < 0.001, ****p < 0.0001, ns; not significant, points without an asterisk are not significant (ns).*

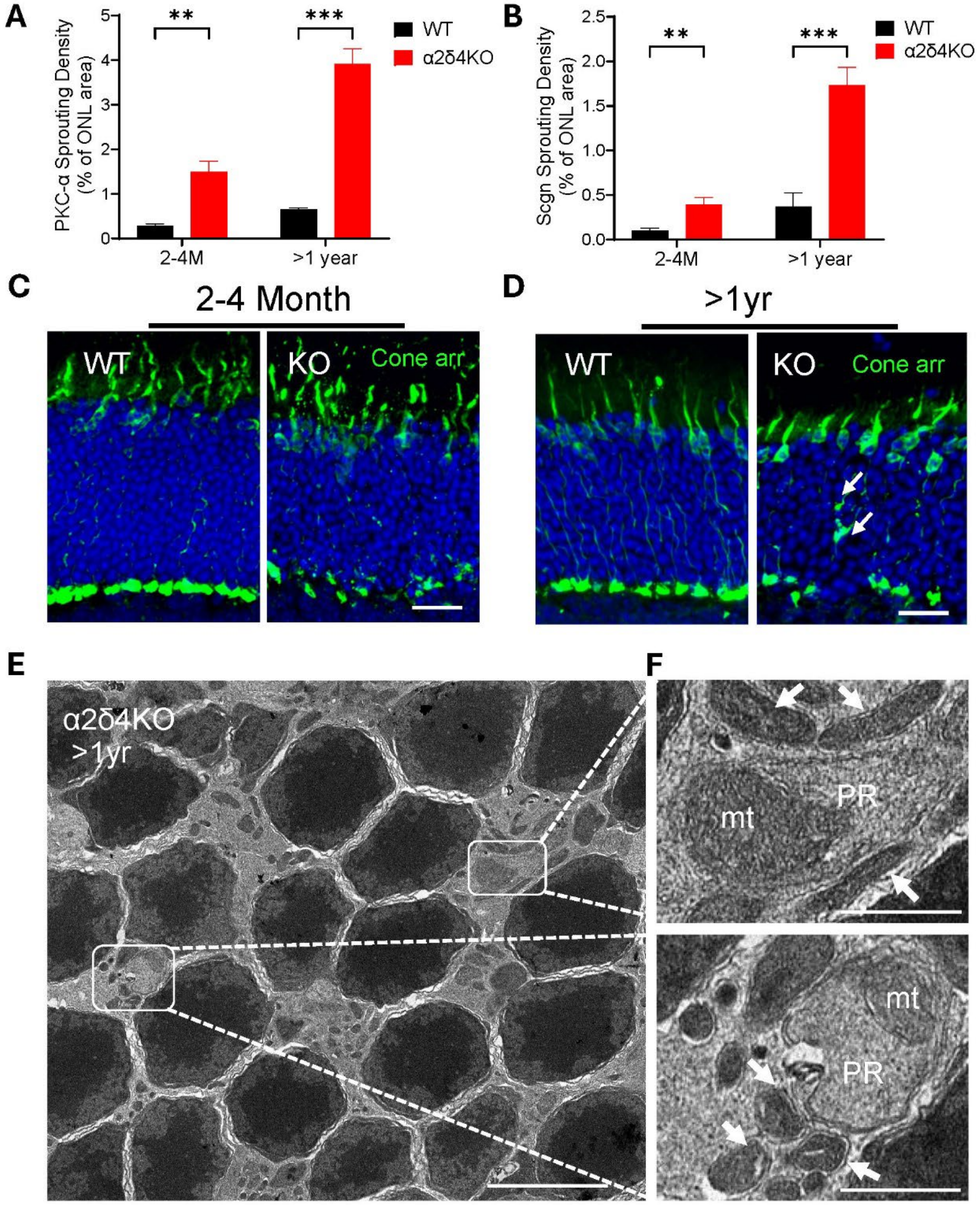

Supplementary Figure 3 (related to Figure 3): Remodeling of bipolar cell dendrites and photoreceptor terminals increases with age in α2δ4KO retina.

A - B. Quantification of the dendritic sprouting area of **A.** rod bipolar cell (RBC) and **B.** cone bipolar cell (CBC) in the ONL normalized to ONL size, in WT and KO mice at 2 – 4 months and >1 year (n = 4 mice per group).

C – D. Confocal images of cone photoreceptors in WT and KO retinal sections stained with anti-cone arrestin at **C.** 2 – 4 months, and **D.** >1 year. Arrows indicate retracted cone pedicles in the ONL. Scale bar, 20 µm.

E. F TEM images of the ONL in >1 year KO retina showing electron-dense double-membrane structures enclosing presumed bipolar dendrites (arrows) adjacent to photoreceptor terminals (PR) containing mitochondria (mt). Scale bar, 4 µm; inset, 1 µm.

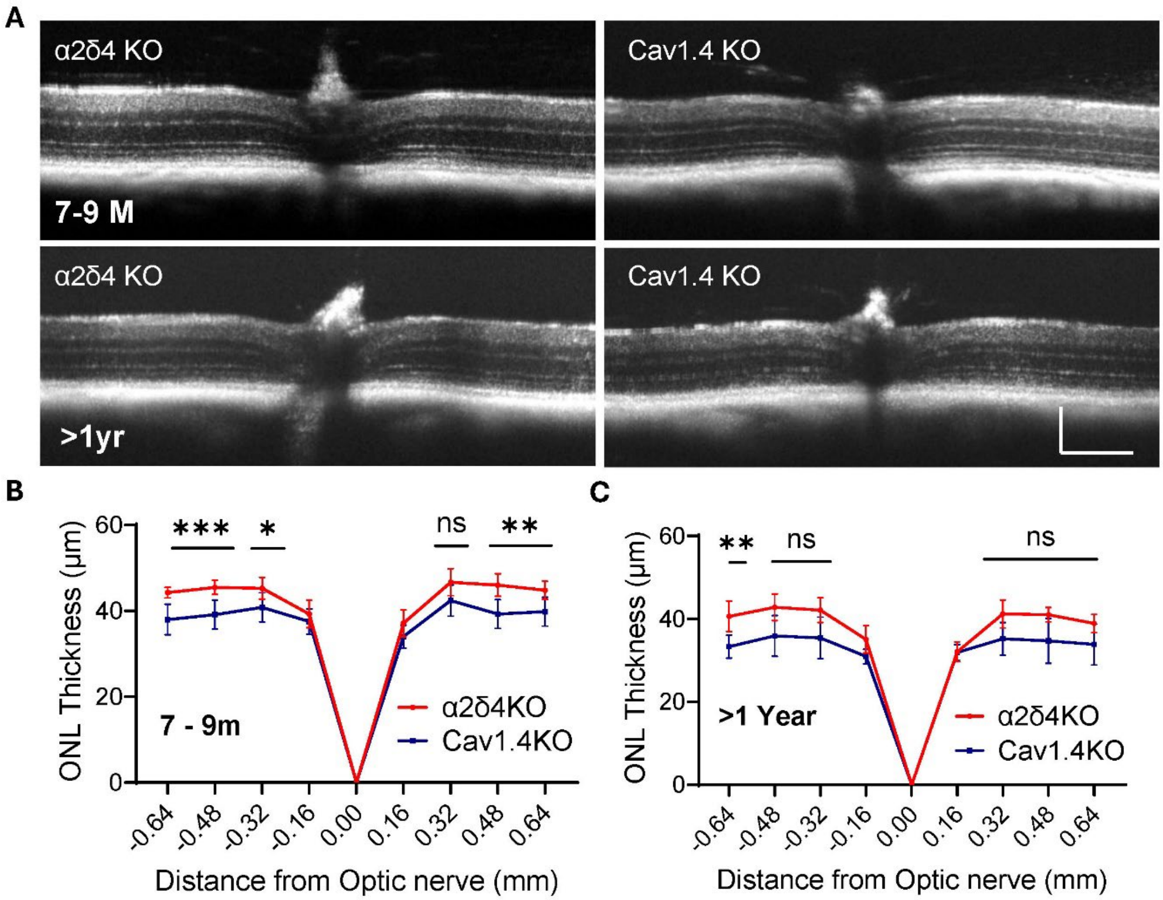

Supplementary Figure 4 (related to Figure 4): OCT imaging reveals progressive ONL thinning in α2δ4KO and Cav1.4KO retinas

A. Representative OCT images of α2δ4KO and Cav1.4KO retina at 7 – 9 months and > 1 year. Scale bar, 100 µm

B – C. Quantification of ONL thickness across retinal eccentricities in α2δ4KO and Cav1.4KO mice at 7 – 9 month and >1 year (n = 8 mice per group).

Error bars are SEM, unpaired t-test. **p < 0.05, **p < 0.01, ***p < 0.001, ns; not significant*

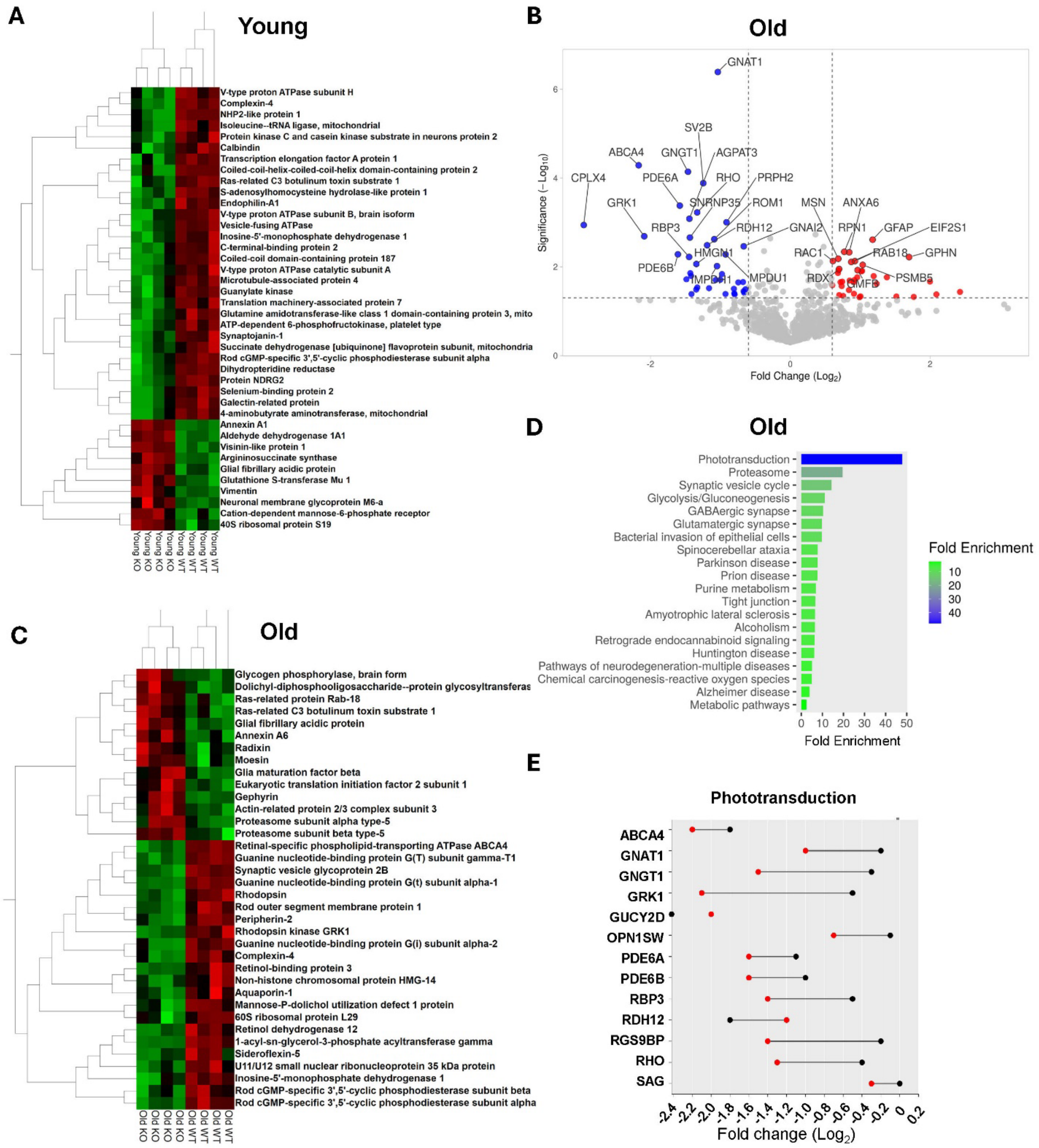

Supplementary Figure 5 (related to Figure 5): Proteomic analysis of differentially expressed proteins in old α2δ4KO retina.

A, C. Heatmap analysis of DEPs in α2δ4KO retinas at **A.** 2 – 4 months (*young*) and **C.** > 1 year (*old*).

B. Volcano plot of DEPs in the old α2δ4KO retinas. DEPs with fold change (FC) ≥ 1.5 are highlighted in blue (downregulated) and red (upregulated). Statistical significance determined by t-test ($p \leq 0.05$); $n = 4$ mice.

D. KEGG pathway enrichment analysis showing the top 20 perturbed pathways in old KO retinas.

E. Dumbbell plot showing expression changes of proteins associated with phototransduction pathway in young *(black dots*) and old (*red dots*) retinas. Statistical significance determined by unpaired *t*-test ($p \leq 0.05$).

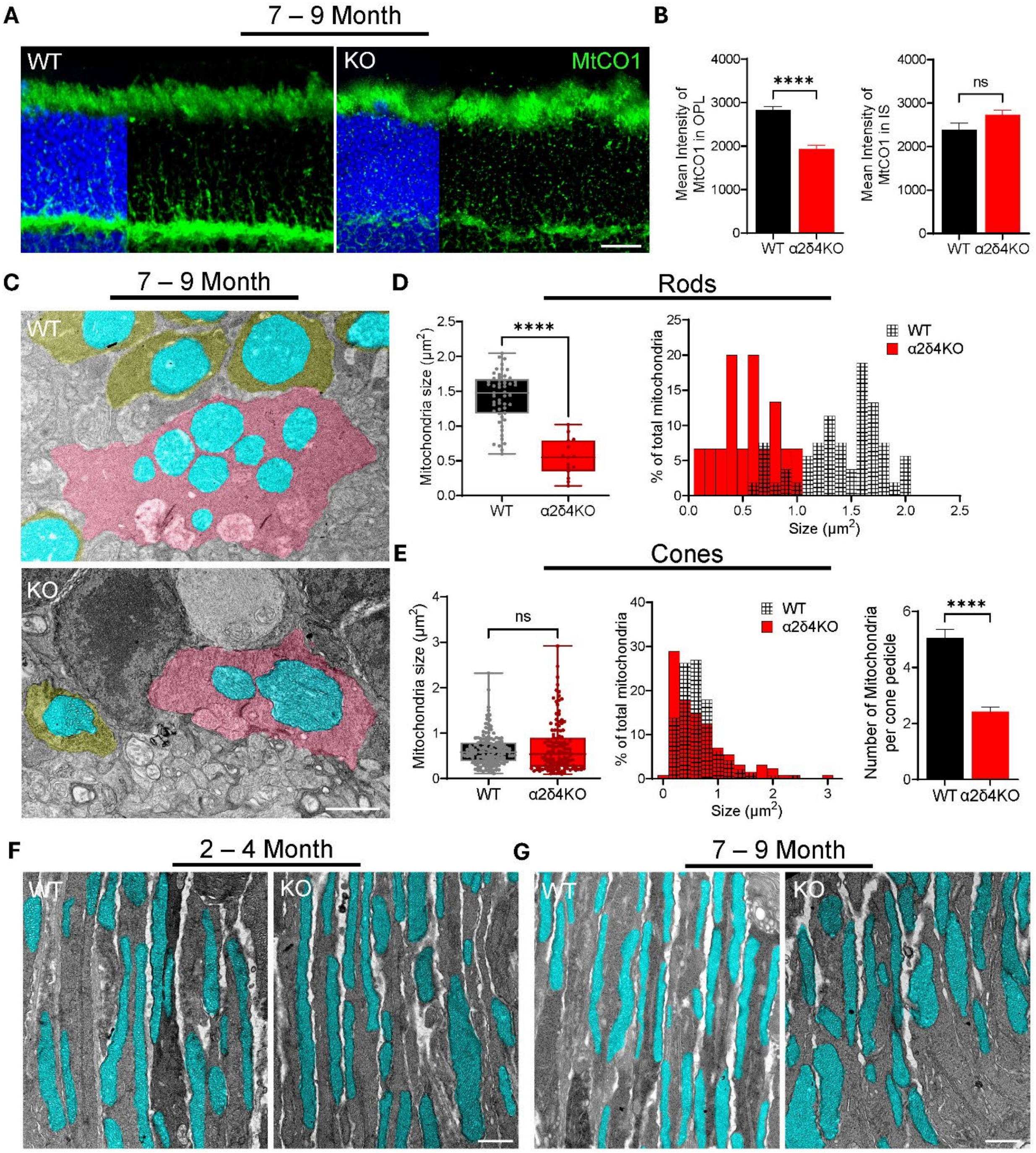
A
7 – 9 Month
WT
KO
MtCO1
B
Mean Intensity of MtCO1 in OPL
****
WT α2δ4KO
Mean Intensity of MtCO1 in IS
ns
WT α2δ4KO
C
7 – 9 Month
WT
KO
D
Rods
Mitochondria size (μm2)
****
WT α2δ4KO
% of total mitochondria
Size (μm2)
WT
α2δ4KO
E
Cones
Mitochondria size (μm2)
ns
WT α2δ4KO
% of total mitochondria
Size (μm2)
WT
α2δ4KO
Number of Mitochondria per cone pedicle
****
WT α2δ4KO
F
2 – 4 Month
WT
KO
G
7 – 9 Month
WT
KO

Supplementary Figure 6 (related to Figure 6). α2δ4KO loss alters mitochondrial content and ultrastructure in photoreceptor terminals but not inner segment.

A. Confocal images of MtCO1-stained retinal sections of WT and KO mice at 7 – 9 months. Scale bar, 50 μm.

B. Quantification of MtCO1 fluorescence intensity in the OPL and IS (n = 4).

C. Representative TEM images showing ultrastructure of rod spherules (*yellow*) and cone pedicles (*pink*) containing mitochondria (*blue*) in WT and KO 7 – 9 months retinas. Scale bar, 1 μm.

D. Quantification of mitochondrial size size (*left*) and occupancy (*right*) in rod spherules of WT and KO retinas at 7 – 9 months (n; WT: 53 rod terminals from 3 mice; KO: 15 terminals from 3 mice).

E. Quantification of mitochondrial size (*left*), occupancy (*middle*) and number (*right*) in cone pedicles of WT and KO retinas at 2 – 4 months (n; WT: 145 cone terminals from 3 mice; KO: 128 terminals from 3 mice).

F – G. Representative TEM images showing inner segment mitochondria (blue) in WT and KO at **F.** 2 – 4 months and **G.** 7 – 9 months. Scale bar, 1 μm.

Error bars are SEM, unpaired t-test. ****$p < 0.0001$, *ns; not significant*

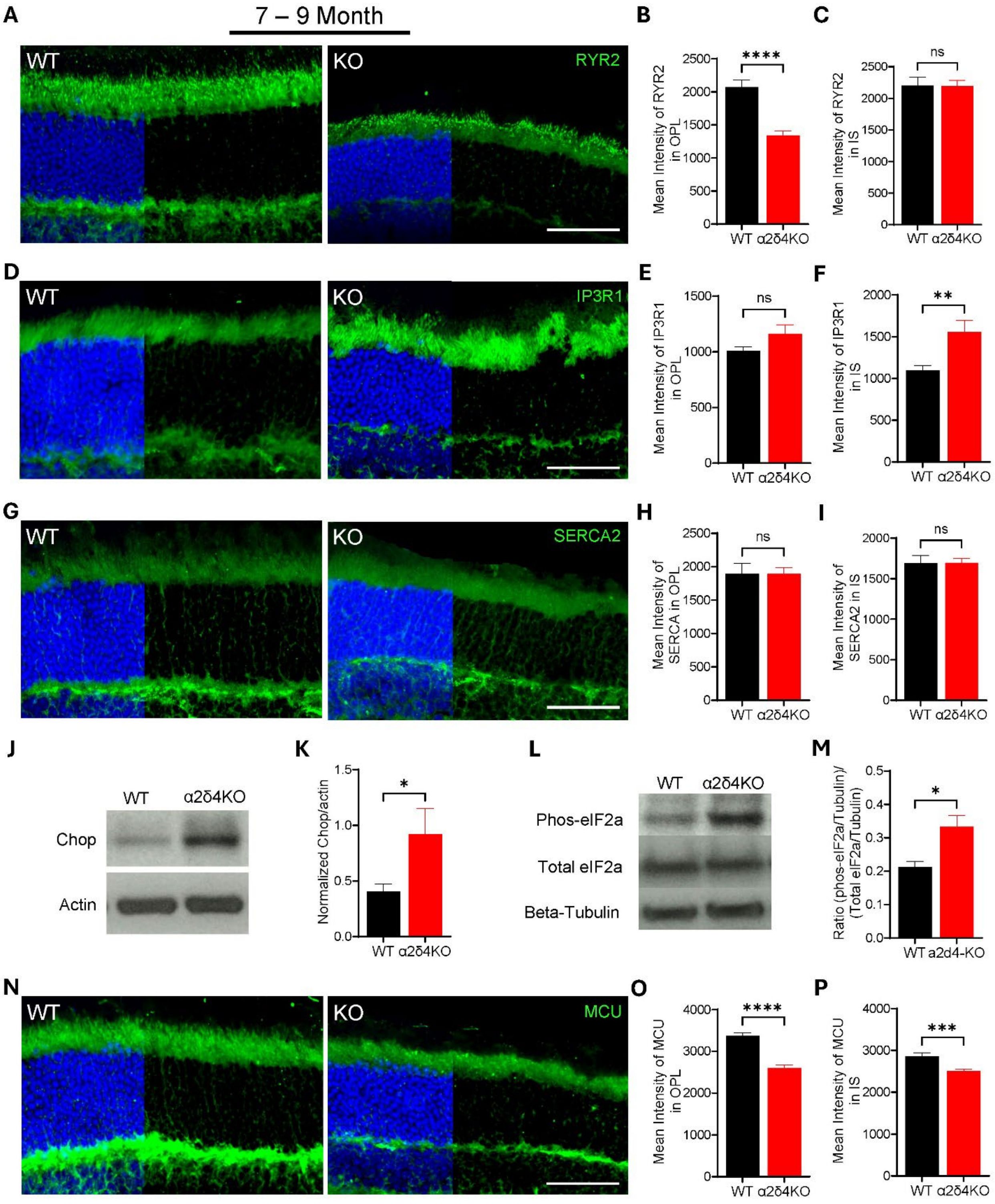

Supplementary Figure 7 (related to Figure 7). α2δ4 loss perturbs ER and mitochondrial Ca^{2+} in photoreceptors.

A. Confocal images of RYR2-stained retina sections of WT and KO mice at 7 – 9 months. Scale bar, 50 µm.

B – C. Quantification of RYR2 immunofluorescence intensity in the **B.** OPL and **C.** IS of WT and KO retinas at 7 – 9 months (n = 4 mice per group).

D. Confocal images of IP3R1-stained retina sections of WT and KO mice at 7 – 9 months. Scale bar, 50 μm.

E – F. Quantification of IP3R1 immunofluorescence in the **E.** OPL and **F.** IS of WT and KO retina at 7 – 9 months (n = 4 mice per group).

G. Confocal images of SERCA2-stained retina sections of WT and KO mice at 7 – 9 months. Scale bar, 50 μm.

H – I. Quantification of SERCA2 immunofluorescence intensity in the **H.** OPL and **I.** IS of WT and KO retina at 7 – 9 months (n = 4 mice per group).

J. Representative western blot image of CHOP in WT and KO retina at 2 – 4 months.

K. Quantification of CHOP protein levels normalized to WT.

L. Representative western blot of phosphorylated (phos) and total eIF2α in WT and KO retinas at 2–4 months.

M. Quantification of phosphorylated eIF2α to total ratio normalized to β-tubulin in WT and KO retinas.

N. Confocal images of MCU-stained retina sections in WT and KO mice at 7 – 9 months. Scale bar, 50 μm.

O – P. Quantification of MCU immunofluorescence intensity in the **K.** OPL and **L.** IS of WT and KO retina at 7 – 9 months (n = 4 mice per group).

Error bars are SEM, unpaired t-test. **$p < 0.05$, **$p < 0.01$, ***$p < 0.001$, ****$p < 0.0001$, ns; not significant*
