# Supplementary material for: Dysfunction of α2δ4 leads to photoreceptor degeneration through disrupted synaptic mitochondria and calcium crosstalk
Source: Cell Death Dis. 2026 Mar 23;17(1):337. doi: 10.1038/s41419-026-08587-3 (PMC13039829; doi:10.1038/s41419-026-08587-3)
Supplement: Supplementary file 2 — Related Manuscript File [file 41419_2026_8587_MOESM2_ESM.pptx]

## Slide 1
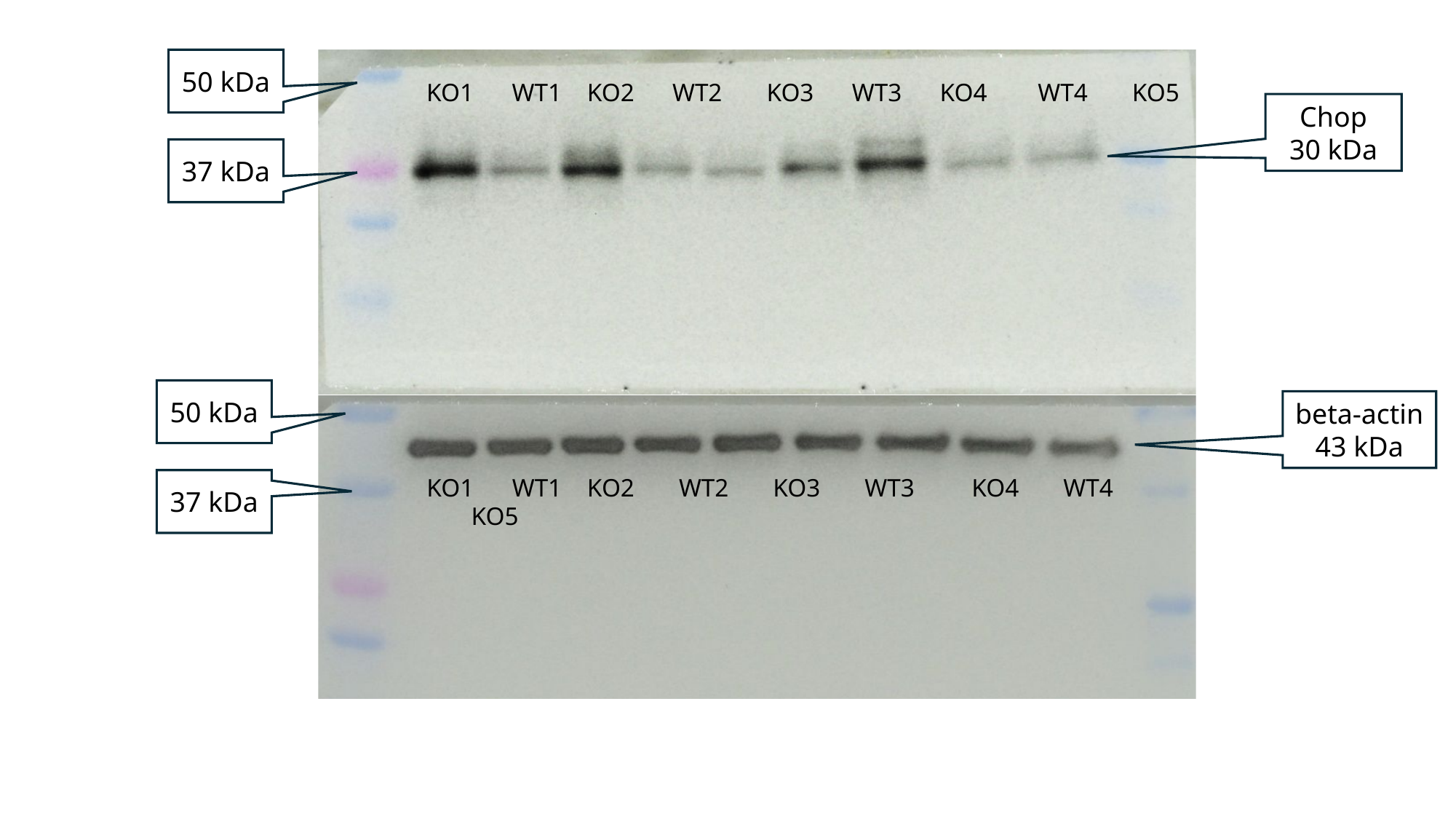

50 kDa
 KO1 WT1 KO2 WT2 KO3 WT3 KO4 WT4 KO5
Chop
30 kDa
37 kDa
50 kDa
beta-actin
43 kDa
 KO1 WT1 KO2 WT2 KO3 WT3 KO4 WT4 KO5
37 kDa

## Slide 2
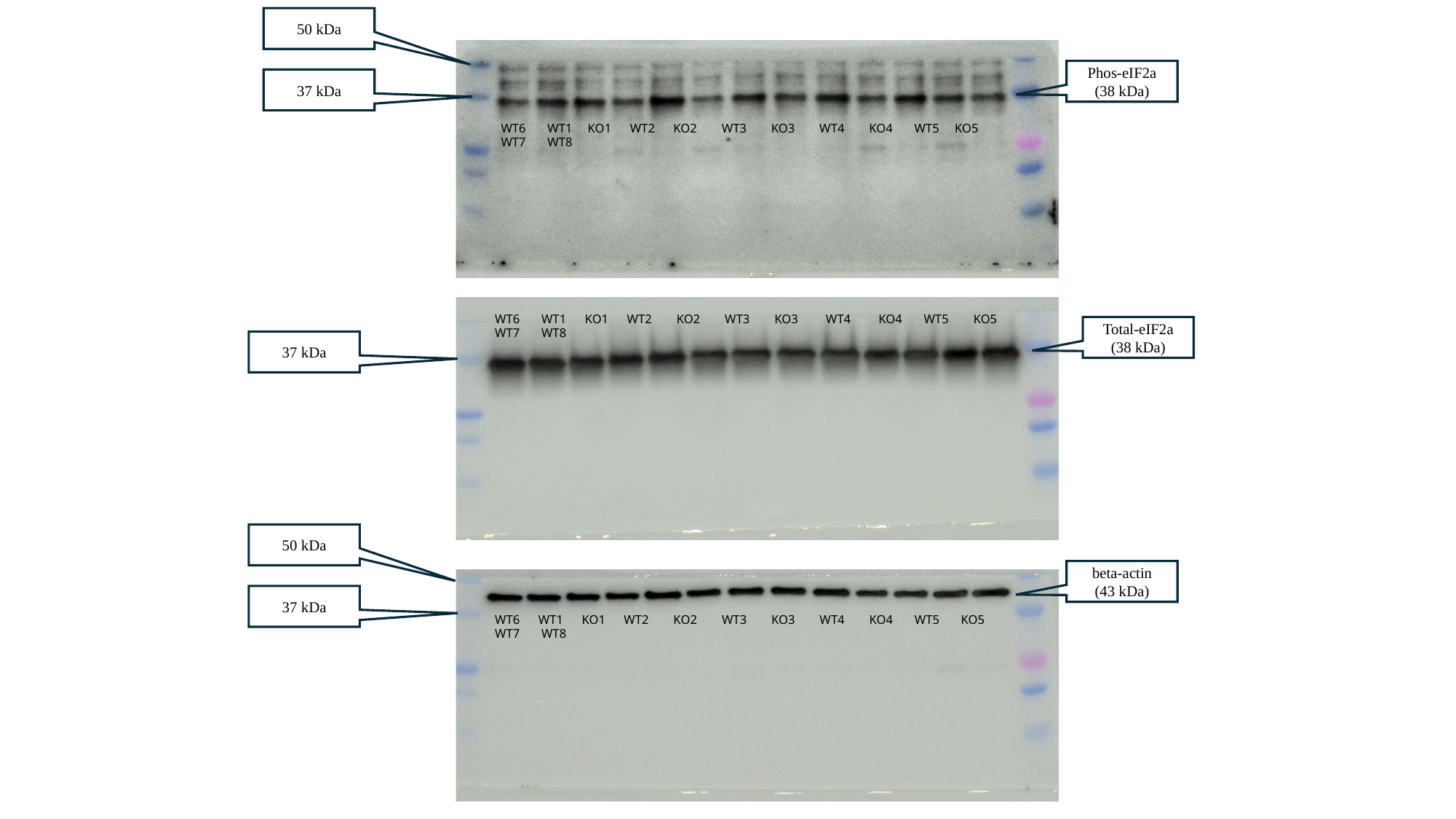

50 kDa
Phos-eIF2a
(38 kDa)
37 kDa
WT6 WT1 KO1 WT2 KO2 WT3 KO3 WT4 KO4 WT5 KO5 WT7 WT8
WT6 WT1 KO1 WT2 KO2 WT3 KO3 WT4 KO4 WT5 KO5 WT7 WT8
Total-eIF2a
(38 kDa)
37 kDa
50 kDa
beta-actin
(43 kDa)
37 kDa
WT6 WT1 KO1 WT2 KO2 WT3 KO3 WT4 KO4 WT5 KO5 WT7 WT8
